# A Protein Thermometer Controls Temperature-Dependent Transcription of Flagellar Motility Genes in *Listeria monocytogenes*

**DOI:** 10.1371/journal.ppat.1002153

**Published:** 2011-08-04

**Authors:** Heather D. Kamp, Darren E. Higgins

**Affiliations:** Department of Microbiology and Immunobiology, Harvard Medical School, Boston, Massachusetts, United States of America; University of Illinois, United States of America

## Abstract

Facultative bacterial pathogens must adapt to multiple stimuli to persist in the environment or establish infection within a host. Temperature is often utilized as a signal to control expression of virulence genes necessary for infection or genes required for persistence in the environment. However, very little is known about the molecular mechanisms that allow bacteria to adapt and respond to temperature fluctuations. *Listeria monocytogenes* (*Lm*) is a food-borne, facultative intracellular pathogen that uses flagellar motility to survive in the extracellular environment and to enhance initial invasion of host cells during infection. Upon entering the host, *Lm* represses transcription of flagellar motility genes in response to mammalian physiological temperature (37°C) with a concomitant temperature-dependent up-regulation of virulence genes. We previously determined that down-regulation of flagellar motility is required for virulence and is governed by the reciprocal activities of the MogR transcriptional repressor and the bifunctional flagellar anti-repressor/glycosyltransferase, GmaR. In this study, we determined that GmaR is also a protein thermometer that controls temperature-dependent transcription of flagellar motility genes. Two-hybrid and gel mobility shift analyses indicated that the interaction between MogR and GmaR is temperature sensitive. Using circular dichroism and limited proteolysis, we determined that GmaR undergoes a temperature-dependent conformational change as temperature is elevated. Quantitative analysis of GmaR in *Lm* revealed that GmaR is degraded in the absence of MogR and at 37°C (when the MogR:GmaR complex is less stable). Since MogR represses transcription of all flagellar motility genes, including transcription of *gmaR*, changes in the stability of the MogR:GmaR anti-repression complex, due to conformational changes in GmaR, mediates repression or de-repression of flagellar motility genes in *Lm*. Thus, GmaR functions as a thermo-sensing anti-repressor that incorporates temperature signals into transcriptional control of flagellar motility. To our knowledge, this is the first example of a protein thermometer that functions as an anti-repressor to control a developmental process in bacteria.

## Author Summary

The ability to sense and respond to environmental changes is essential for the survival of all living organisms. Thermosensors are cellular components that can transform temperature changes into significant cellular responses necessary for adaptation and survival. In this study, we identify a protein thermosensor, GmaR, in the human bacterial pathogen *Listeria monocytogenes* that senses the transition from ambient to human body temperature and transforms this temperature signal into changes that affect bacterial motility and pathogenesis. Bacterial motility is mediated by the production and rotation of long tail-like structures known as flagella that are found on the surface of bacterial cells. Flagellar motility is important for bacterial survival in the environment, but inside a human host, flagella are recognized as a danger signal by the human immune defense system. Temperature-dependent conformational changes in GmaR control the temperature-responsive ON/OFF switch for gene expression required for flagellar motility. This thermo-sensing mechanism aids *L. monocytogenes* pathogenesis by turning OFF flagellar motility genes upon entering a mammalian host, and is important for bacterial survival in the external environment by turning ON flagellar motility in response to ambient temperatures where flagellar motility is needed for nutrient acquisition and colonization of surfaces.

## Introduction

Temperature is an important environmental condition to which organisms must adapt. The most universal and well-studied temperature responsive system is the heat shock response, which protects organisms from sudden stress-inducing increases in environmental temperature (reviewed in [1], [2]). However, even for the heat shock response, the molecular mechanisms for temperature sensing are not fully understood. Microorganisms have not only evolved to sense and react to stress-inducing temperature fluctuations, but also to utilize thermo-sensing components to regulate processes required for adaptation to milder temperature fluctuations. For example, many bacterial pathogens sense mammalian physiological temperature (37°C) and respond via transcriptional and translational changes that have global effects on bacterial physiology, survival, and virulence. These changes result in up-regulation of determinants required for infection of the host and down-regulation of determinants specifically required for extracellular survival.

Thermosensors composed of DNA, RNA, and protein, have been identified in bacteria (reviewed in [3], [4]). These biological thermometers incorporate temperature signals into transcriptional and translational responses required for bacterial adaptation and survival. A DNA thermometer typically involves specific DNA sequences (usually AT rich) that alter DNA structure and curvature in response to temperature. When these temperature-sensitive DNA sequences are strategically located within promoter regions, the binding of regulatory proteins and RNA polymerase is affected and can result in temperature-dependent transcriptional responses [5], [6], [7], [8]. RNA thermometers often act post-transcriptionally by either inhibiting or enhancing translation (reviewed in [9]). The most common RNA thermometer involves a thermo-sensitive, *cis*-acting sequence whose three-dimensional structure occludes ribosome binding to the Shine-Dalgarno sequence at low temperatures, the melting of this sequence at high temperatures permits translation [10], [11], [12], [13]. Trans-acting small non-coding RNAs also exist that have been shown to enhance translation [14], [15]. The most diverse group of thermosensors in biological systems are the protein thermometers. These protein-based thermosensors include DNA-binding transcriptional regulators, protein binding chaperones, proteases, sensor kinases, and methyl-accepting chemotaxis proteins (reviewed in [3]). Due to the functional diversity of thermo-sensing proteins, the direct downstream effects are also diverse, affecting transcription, signal transduction, protein stability and proteolysis.

The transition from ambient temperature (22°C–28°C) to host physiological temperature (37°C) is an important signal that bacterial pathogens sense upon transitioning from an environmental reservoir or vector to a warm-blooded host. Many pathogens up-regulate virulence genes in response to temperature using thermosensors (reviewed in [16]). For example, in *Shigella flexneri, Salmonella enterica,* and *Escherichia coli*, temperature-dependent changes in DNA topology alter DNA binding of the nucleoid-associated protein H-NS, which represses virulence genes at low temperatures [7], [8], [17]. *Yersinia* species also regulate virulence genes in response to temperature, though the mechanisms are poorly understood and complex; they involve several thermo-sensing components [10], [18], [19], [20], [21]. In the Lyme disease spirochete *Borrelia burgdorferi,* an unusual trans-acting RNA thermometer enhances rather than inhibits translation of the alternative sigma factor RpoS [15], which has a key role in the regulation of the virulence-associated major outer surface proteins required for host infection [22]. *Bordetella pertussis* also regulates virulence genes in a temperature-dependent manner, presumably through the BvgAS two-component regulatory system, however the thermo-sensing mechanisms have yet to be identified [23]. Finally, in *L. monocytogenes* (*Lm*), an RNA thermometer located in the 5′ untranslated region of transcripts for the virulence regulator PrfA controls temperature-dependent translation of PrfA, which results in temperature-dependent transcription of PrfA-regulated virulence genes [12].

For many facultative bacterial pathogens, flagellar motility is important for survival outside of the host, often playing a critical role in nutrient acquisition through chemotaxis and is required for biofilm formation, which aids in bacterial persistence in the environment (Reviewed in [24], [25]). Flagellar motility is also important for colonization of the host during the early stages of infection by enhancing bacterial adherence to and invasion of host cells [26], [27], [28]. However, flagella are immunostimulatory and deleterious for bacterial survival inside the host since they are recognized by both the human TLR5 and Ipaf receptors [29], [30], [31]. Therefore, some bacterial pathogens adapt to the host environment by repressing flagellar motility at physiological temperatures once colonization has been established [32], [33], [34], [35]. While a few virulence gene-regulating thermosensors have been identified in bacterial pathogens (reviewed above), here we report the first thermo-sensing mechanism controlling the ON/OFF switch for flagellar motility in a human pathogen in response to physiological temperature. We have identified a protein thermometer in *Lm* that controls temperature-dependent transcription of flagellar motility genes.

In *Lm,* flagellar motility is important for colonization of surfaces both inside and outside of the host [27], [36] and is temperature-dependent [35]. *Lm* is flagellated and motile at ambient temperatures (22°C–28°C), and is non-flagellated and non-motile at mammalian physiological temperature (37°C) [35]. Temperature-dependent transcription of flagellar motility genes is controlled by the reciprocal activities of the MogR repressor and the GmaR anti-repressor, and requires the DegU response regulator [37], [38], [39], [40]. MogR represses flagellar motility gene transcription at 37°C by binding to all flagellar motility gene promoters [37], [39]. While MogR is constitutively expressed at all temperatures [39], at temperatures below 37°C the MogR anti-repressor, GmaR, directly antagonizes MogR repression activity [38]. Temperature-dependent expression of GmaR restricts transcription of flagellar motility genes to low temperatures [38]. While the DegU response regulator constitutively activates transcription of *gmaR* in a temperature-independent manner [40], we recently determined that a post-transcriptional mechanism limits GmaR protein production to low temperatures [40]. Since MogR represses the transcription of all flagellar motility genes, production of the GmaR anti-repressor at low temperatures is the first committed step for flagellar motility. Transcription of *gmaR* is also MogR-repressed and is therefore up-regulated by GmaR anti-repression activity [40]. In this study, we determined the temperature-dependent post-transcriptional mechanism required for transcription of flagellar motility genes in *Lm*. We demonstrate that the GmaR anti-repressor is also a protein thermometer that undergoes a conformational change in response to temperature that affects its binding interaction with the MogR repressor. Destabilization of the MogR:GmaR complex at host physiological temperature (37°C), releases MogR to repress transcription of *gmaR* along with all flagellar motility genes, while free GmaR is degraded. Conversely, stabilization of the MogR:GmaR anti-repression complex at low temperatures results in de-repression of flagellar motility genes and production of flagella. Thus, our findings indicate that GmaR is a thermo-sensing anti-repressor that confers temperature specificity to flagellar motility gene transcription in *Lm*.

## Results

### GmaR is degraded in a temperature-dependent manner and in the absence of MogR

**Figure 1:**
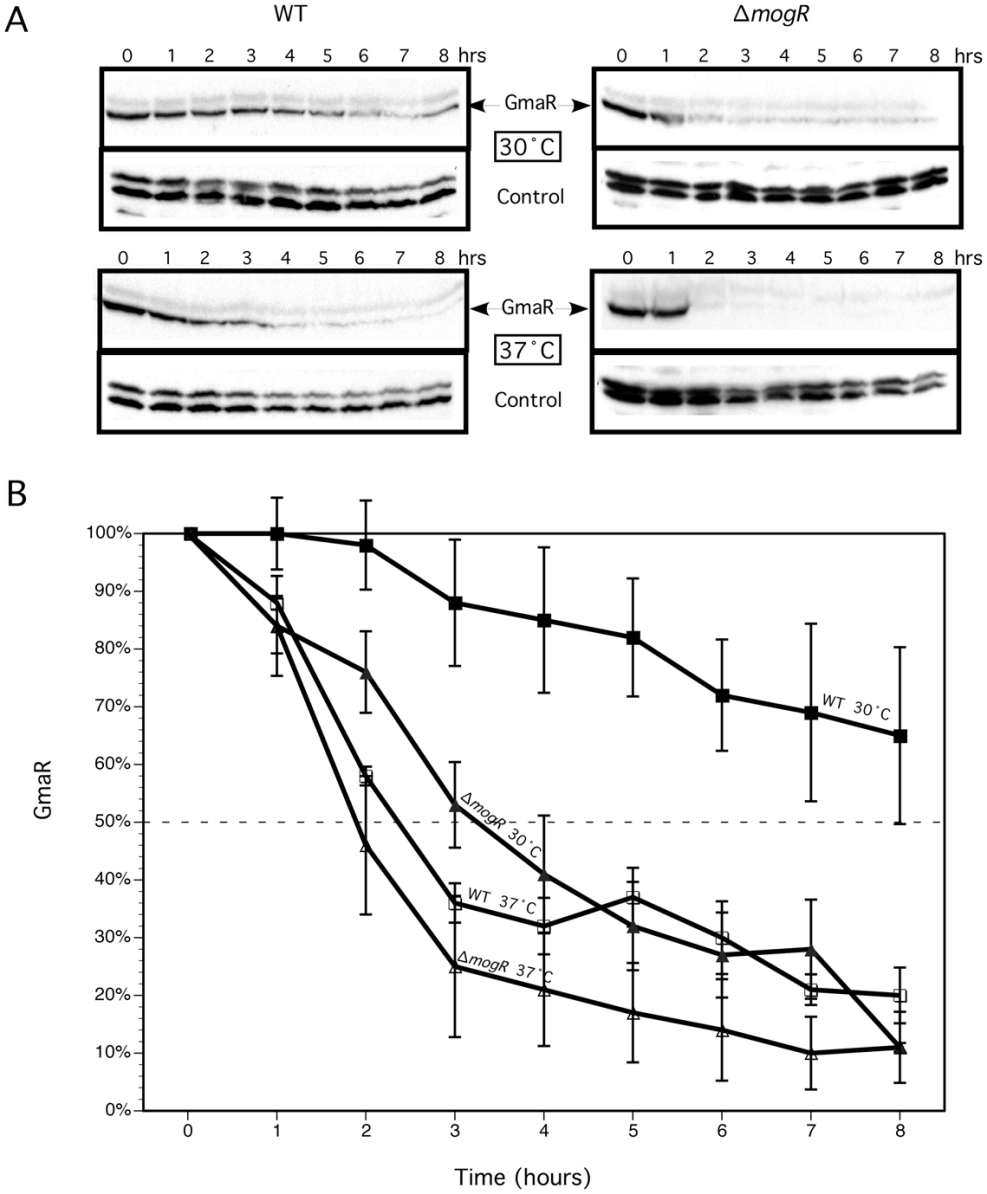
GmaR protein is degraded at 37°C and in the absence of MogR. GmaR protein levels were determined following growth in the presence of tetracycline and upon temperature shift. Wild-type (WT) and Δ*mogR* bacteria were grown 16–18 h at room-temperature (RT: 22°C–24°C) without shaking. Cultures were diluted to an OD_600_ = 0.4, split into two cultures and 8 µg/ml tetracycline was added. Cultures were then shifted to either 30°C or 37°C and grown without shaking for an additional 8 h. Samples were taken every hour for determination of OD_600_, cfu/mL (Figure S1) and Western blot analysis. (**A**) Western blot analysis of GmaR and DegU (control). (**B**) Quantitative analysis of Western blots. Densitometry of Western blots from three independent experiments as described above was performed. For each condition, the percentage of GmaR protein present at each time point following the addition of tetracycline in relation to GmaR present at T = 0 is shown. The intersection of the dashed line represents the time at which 50% of the initial GmaR protein was present. Data represent the means and standard deviations of three independent experiments.

Production of the GmaR anti-repressor is the first committed step for flagellar motility at low temperatures. We have previously demonstrated that *gmaR* transcripts are stable at elevated temperatures [40]; therefore, we hypothesized that the post-transcriptional mechanism that limits GmaR production to low temperatures involves either translational control or protein stability. To distinguish between these two possibilities, we analyzed GmaR protein levels following temperature shift to 30°C or 37°C in the presence of the translational inhibitor tetracycline. The concentration of tetracycline used (8 µg/ml) resulted in bacterial growth arrest, but not cell death, as determined by bacterial growth and viability analyses (Figure S1). Bacteria were grown without shaking at room temperature (RT: 22°C–24°C), a condition that allows for maximum GmaR expression, prior to temperature shift and antibiotic treatment. Western blot analysis was used to determine GmaR protein levels in wild-type and Δ*mogR* bacteria over an 8 h period following temperature shift. Results demonstrated that both temperature and the absence of MogR affected GmaR protein stability (Figure 1A). Densitometry from three independent experiments was used for quantitative analysis of GmaR (Figure 1B). The GmaR protein levels at each time point were normalized to the amount of GmaR protein at T = 0 for each condition. For wild-type bacteria grown at 30°C, a temperature permissive for flagellar motility, the half-life of GmaR was determined to be >8 h (Figure 1B). In contrast, for Δ*mogR* bacteria grown at 30°C, the apparent half-life of GmaR was reduced to ∼3 h. When both wild-type and Δ*mogR* bacteria were grown at 37°C, the half-life of GmaR was greatly reduced to ∼2–2.5 h (Figure 1B). Therefore, these studies revealed that the stability of GmaR is temperature-dependent in wild-type bacteria and that degradation of GmaR is more rapid in the absence of MogR at both 30°C and 37°C, suggesting that MogR contributes to the stability of GmaR.

### The interaction between GmaR and MogR is temperature-sensitive

GmaR and MogR directly interact to form a protein-protein complex *in vitro* and this interaction is required to relieve MogR repression of flagellar motility gene transcription at low temperatures [38]. Since GmaR was degraded more rapidly at 30°C within Δ*mogR* bacteria compared to wild-type bacteria (Figure 1), we hypothesized that MogR may function as a chaperone for GmaR, where the MogR:GmaR anti-repression complex stabilizes GmaR and prevents proteolysis of GmaR at temperatures below 37°C. However, the interaction between MogR and GmaR is predicted to be temperature-sensitive, as MogR binds flagellar promoter DNA at elevated temperatures (37°C and above) to repress transcription of flagellar motility genes [37], [39]. Therefore, we determined whether temperature affects the binding affinities of MogR to GmaR and/or MogR to flagellar promoter region DNA at 30°C and 37°C.

**Figure 2:**
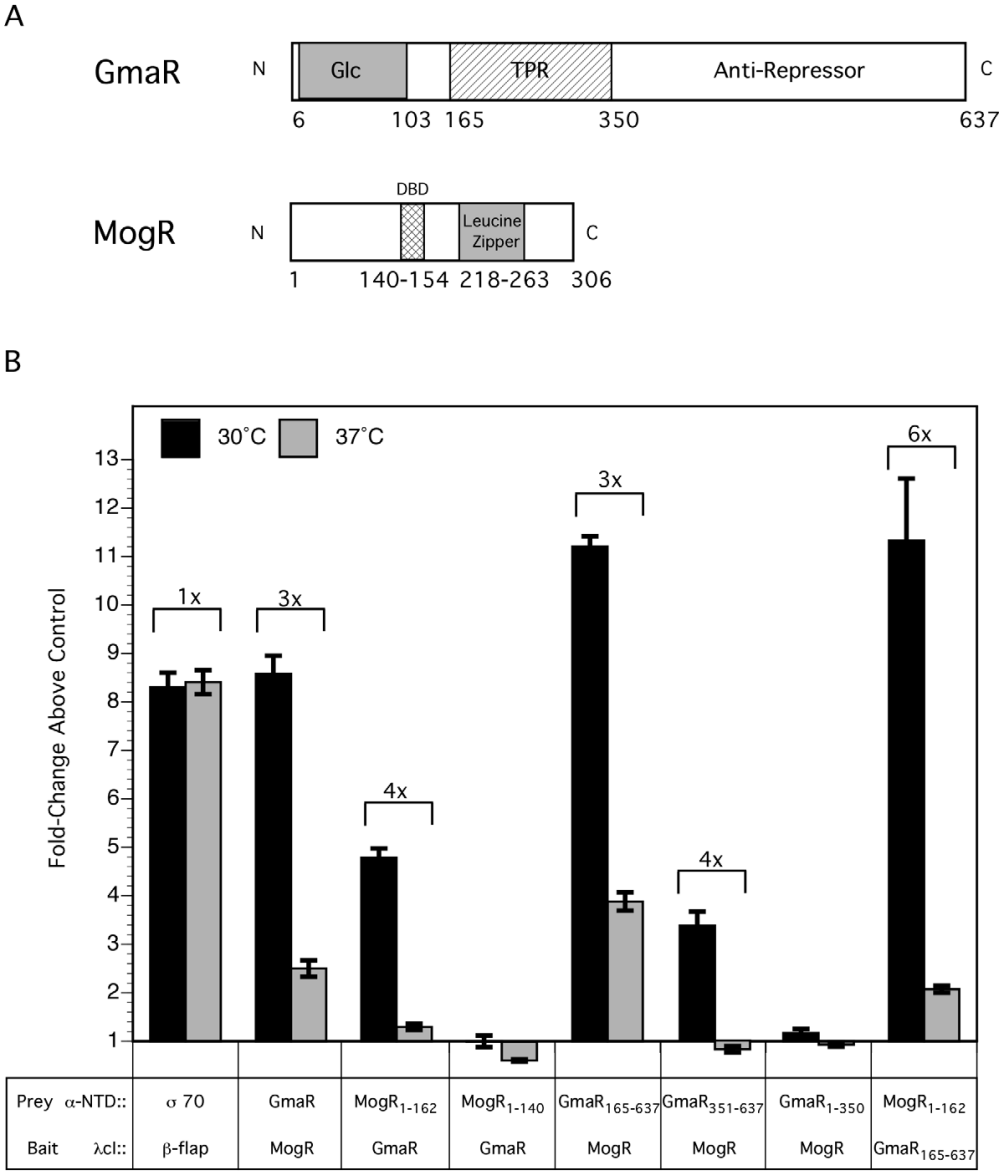
The interaction between GmaR and MogR is temperature-sensitive. (**A**) Schematic representation of full-length GmaR and MogR proteins. The predicted glycosyltransferase (Glc) domain (amino acids 6–103) of GmaR is shaded in grey. Three tetratricopeptide repeat (TPR) domains (amino acids 165–350) of GmaR are represented as a hatched box. The MogR-binding anti-repressor domain of GmaR (amino acids 351–637) is labeled. The DNA binding domain (DBD) of MogR (amino acids 140–154) is indicated as a hatched box. The predicted leucine zipper domain (amino acids 218–263) of MogR is shaded in grey. (**B**) The *E. coli* two-hybrid assay results reported as fold-change above the negative control. Black bars represent the interaction at 30°C and grey bars 37°C. The difference in fold-change between the two temperatures is indicated above each interaction. Data represent the means and standard deviations of three independent experiments performed on the same day. Assays were performed on three separate days with similar results.

Using an *E. coli* two-hybrid system, we analyzed the effect of temperature on the interaction between GmaR and MogR. In this assay, contact between one protein fused to the α-amino terminal domain (α-NTD) of RNA polymerase (prey) and a second protein fused to the DNA-bound λcI protein (bait), activates transcription of a *lacZ* reporter gene under control of a promoter bearing an upstream λ operator site [41]. Specifically, we made α-NTD and λcI fusions to both GmaR and MogR (Figure 2A). In addition to the full-length proteins, we also analyzed MogR_1-162_, which lacks the leucine zipper motif; MogR_1-140_, which lacks both the leucine zipper motif and the DNA binding domain (DBD) [42]; GmaR_165-637_, which lacks the N-terminal glycosyltransferase domain; GmaR_351-637_, which lacks both the glycosyltransferase domain and the tetratricopeptide repeat region (TPR); and GmaR_1-350_, which lacks the C-terminal anti-repressor domain of GmaR (A. Shen and D. Higgins, unpublished), but contains the glycosyltransferase domain and TPR region (Figure 2A). As a positive control, the λcI protein was fused to the β-flap subunit of RNA polymerase (β-flap), and the α-NTD was fused to σ^70^ region 4, which have previously been shown to directly interact [43]. β-galactosidase activity was determined at both 30°C and 37°C for each bait:prey pair and reported in Miller units (Figure S2A, black bars). As a negative control, the α-NTD was included without a fusion protein and analyzed for interaction with each λcI protein fusion to establish a background level of β-galactosidase activity (Figure S2A, grey bars). For each bait:prey pair at each temperature, the fold-change above the negative control was plotted and the difference in the fold-change between the two temperatures was indicated (Figure 2B). There was no detectable difference at the two temperatures in the binding interaction of the positive control between the β-flap and σ^70^ (Figure 2B). However, temperature-dependent differences were observed with the interaction between MogR and GmaR. Specifically, all of the MogR and GmaR fusion proteins which demonstrated an interaction showed at least a 3-fold stronger interaction at 30°C than 37°C (Figure 2B), indicating that the MogR:GmaR complex is affected by temperature. The interaction between full-length GmaR and full-length MogR or MogR_1-162_ was 3–4 fold stronger at 30°C than at 37°C. Consistent with the observation that the MogR:GmaR binding interaction requires the DNA binding motif, but not the leucine zipper motif of MogR (A. Shen and D. Higgins, unpublished), the MogR_1-140_ truncation did not interact with full-length GmaR in the two-hybrid assay (Figure S2A and Figure 2B). The GmaR_165-637_ and the GmaR_351-637_ truncations, which lack the glycosyltransferase or the glycosyltransferase and TPR repeat regions respectively, also resulted in a 3–4 fold stronger interaction with full-length MogR at 30°C than at 37°C. In contrast, GmaR_1-350_, which lacks the predicted anti-repressor domain, did not interact with MogR (Figure S2A and Figure 2B). These results reinforce the observation that formation of the MogR:GmaR complex does not require either the glycosyltransferase domain or the TPR repeat region of GmaR and that these domains do not confer temperature specificity. Furthermore, Western blot analysis of the fusion proteins in *E. coli* revealed that both GmaR and MogR were stable at both temperatures (Figure S2B). The *E. coli* two-hybrid results suggest that either GmaR or MogR may be undergoing a temperature-dependent conformational change that affects the binding interaction between the anti-repressor and repressor.

**Figure 3:**
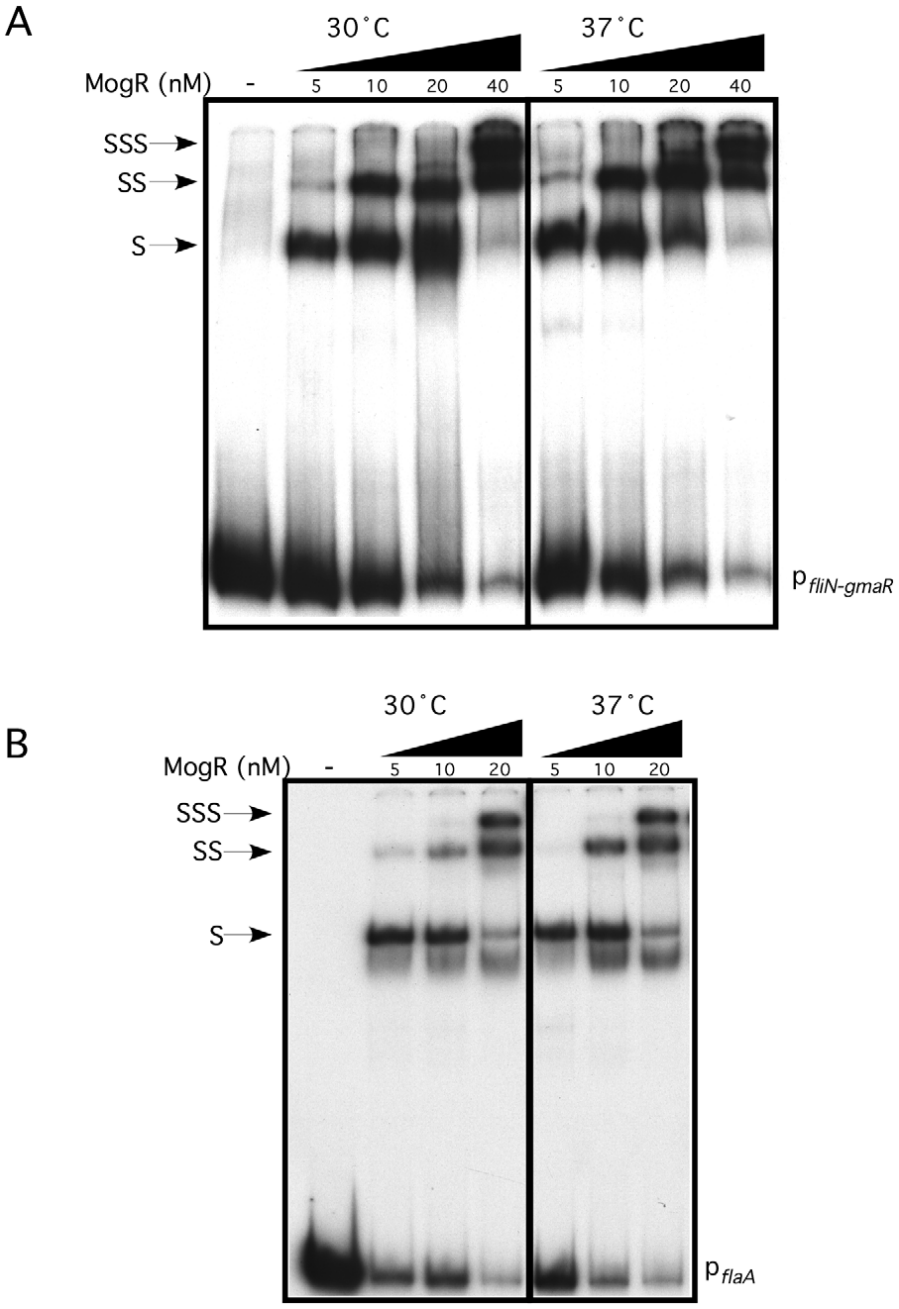
MogR binds to flagellar gene promoter region DNA in a temperature-independent manner. Gel mobility shift analysis of MogR binding to *fliN-gmaR* (**A**) and *flaA* (**B**) promoter region DNA. Radiolabeled promoter region DNA fragments were incubated at either 30°C or 37°C with increasing concentrations of MogR-His_6_. Binding reactions were separated by non-denaturing PAGE and detected by autoradiography. Shifted (S), supershifted (SS), and super-supershifted (SSS) DNA complexes are indicated.

**Figure 4:**
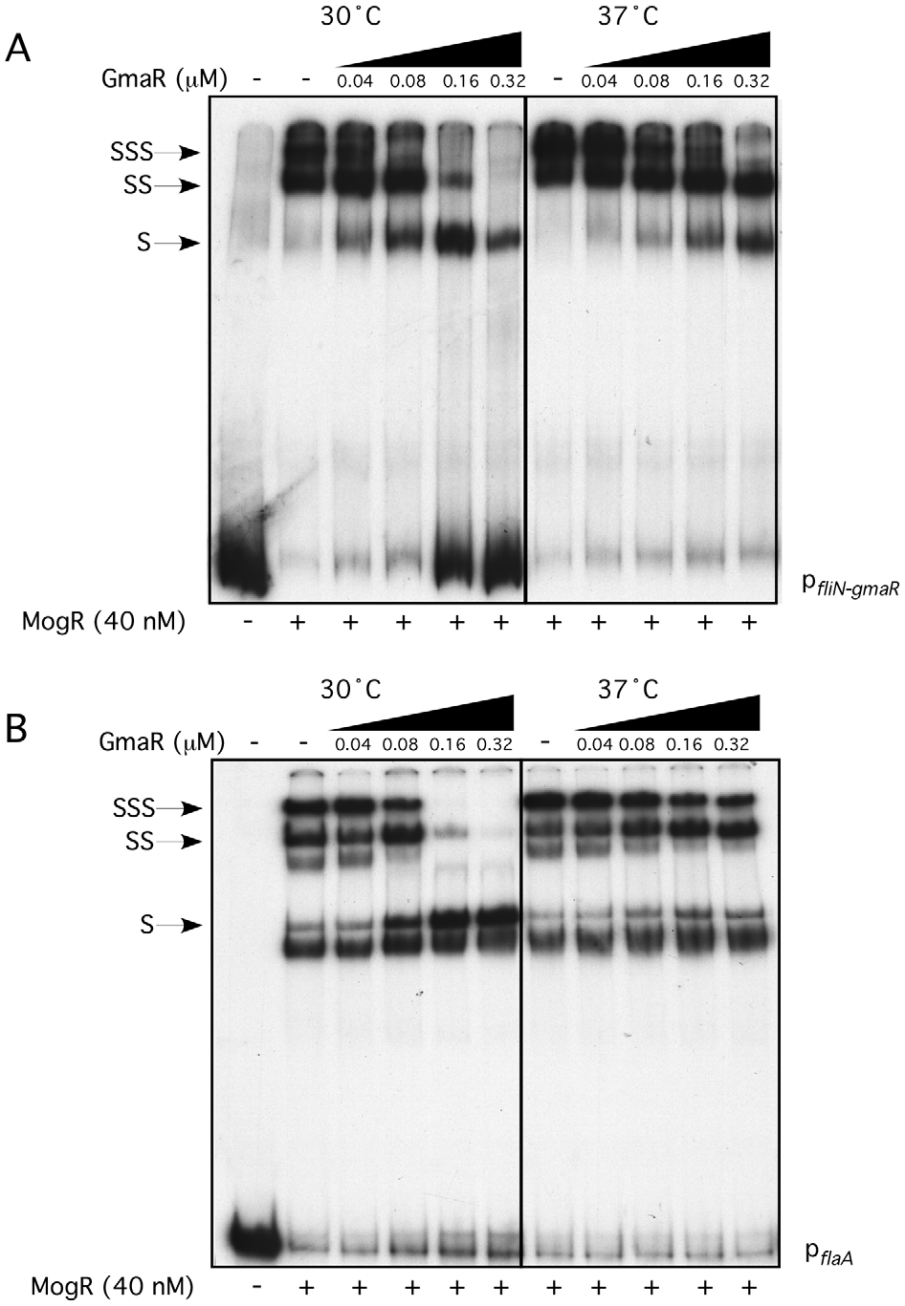
Formation of the MogR:GmaR complex is more efficient at 30°C than at 37°C. Gel mobility shift analysis of MogR binding to *fliN-gmaR* (**A**) and *flaA* (**B**) promoter region DNA. Radiolabeled promoter region DNA fragments were incubated at either 30°C or 37°C with 40 nM MogR-His_6_ for 30 min. GmaR-His_6_ was then added at increasing concentrations and incubated for an additional 30 min at the indicated temperature. Binding reactions were separated by non-denaturing PAGE and detected by autoradiography. Shifted (S), supershifted (SS), and super-supershifted (SSS) DNA complexes are indicated.

Our prior studies determined that MogR binds and shifts flagellar promoter region DNA in a gel mobility shift assay performed at 30°C and that GmaR antagonizes MogR DNA binding activity through a direct protein-protein interaction [38], [39]. Based on the two-hybrid data indicating that the MogR:GmaR interaction is temperature-sensitive in *E. coli* (Figure 2 and Figure S2), we further examined the effect of temperature on both the MogR:GmaR complex and MogR:DNA complexes using gel mobility shift analysis at both 30°C and 37°C. To determine if MogR may undergo a temperature-dependent conformational change, we first analyzed the ability of purified MogR to bind either *fliN-gmaR* or *flaA* promoter region DNA (p*_fliN-gmaR_* or p*_flaA_*, respectively) using gel mobility shift analysis at both 30°C and 37°C. MogR bound both p*_fliN-gmaR_* (Figure 3A) and p*_flaA_* (Figure 3B) with equal affinity at both temperatures, indicating that both MogR and the promoter region DNA were not affected by temperature. We next determined the effect of adding increasing concentrations of GmaR to MogR previously bound to flagellar promoter region DNA at either 30°C or 37°C. The addition of GmaR to the pre-formed MogR:DNA complexes resulted in a rapid decrease in shifted MogR:DNA complexes at 30°C, presumably due to MogR:GmaR complex formation (Figure 4A and 4B, 30°C). However, at 37°C greater than 2-fold more GmaR was required to achieve similar decreases in shifted MogR:DNA complexes as seen at 30°C (Figure 4A and 4B, 37°C). Furthermore, even higher amounts of GmaR were required to disrupt MogR:p*_flaA_* complexes (Figure 4B) than to disrupt the MogR:p*_fliN-gmaR_* complex at 37°C (Figure 4A). This is likely due to the increased number of MogR binding sites located in the *flaA* promoter region compared to the *fliN-gmaR* promoter region (Figure S3). Since the MogR:DNA complex was unaffected by temperature alone (Figure 3), the differences in the shifted complexes observed at 30°C and 37°C when GmaR was added is likely due to conformational changes in GmaR that affect the interaction between MogR and GmaR. It should be noted that the temperature-dependent decrease in the MogR:GmaR interaction is not due to GmaR degradation since purified GmaR was completely stable at 37°C for the duration of the experiment (data not shown). These results indicate that the MogR:GmaR complex is occurring more often at 30°C than at 37°C and that GmaR is a more effective anti-repressor at 30°C than at 37°C *in vitro*.

### GmaR undergoes a temperature-dependent conformational change

**Figure 5:**
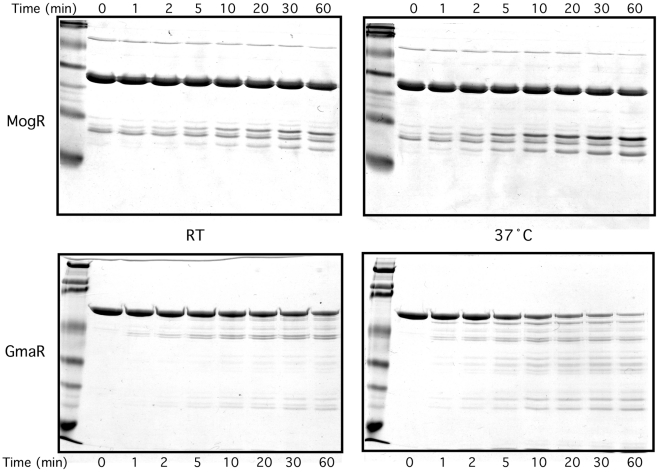
Limited proteolysis of GmaR and MogR with chymotrypsin indicates a temperature-dependent conformational change in GmaR, but not MogR. Purified GmaR-His_6_ or MogR-His_6_ was incubated with chymotrypsin (5000∶1) for 60 min at either RT or 37°C. Reactions were stopped at the sample times indicated by removing 5 µg of protein and mixing with 2X loading buffer. Samples were run on an SDS-PAGE gel and stained with Coomassie stain.

While the *E. coli* two-hybrid results indicated that a temperature-dependent conformational change occurs in either MogR or GmaR (Figure 2 and Figure S2), the gel mobility shift analyses suggested that GmaR undergoes a temperature-dependent conformational change that affects its binding interaction with MogR (Figure 3 and Figure 4). To determine if GmaR undergoes a conformational change upon temperature shift from RT to 37°C, we performed limited proteolysis of purified GmaR over the course of 1 h at both RT and 37°C using chymotrypsin. As a control, purified MogR was similarly treated. The proteolysis of purified MogR at both temperatures was comparable with no significant difference observed in the banding pattern or rate of digestion (Figure 5, upper panel). This result correlates with data indicating that DNA binding by MogR, and by inference its protein conformation, is not affected by a temperature shift to 37°C (Figure 3A and 3B). However, limited proteolysis of purified GmaR resulted in more abundant and unique degradation products at 37°C as compared to RT (Figure 5, lower panel). This result indicates that the protein conformation of GmaR is different at 37°C than at RT, and therefore temperature may induce a conformational change in GmaR. Similar results were observed when both MogR and GmaR were treated with trypsin instead of chymotrypsin, which cleaves proteins at different amino acid residues to yield a different banding pattern (Figure S4). Therefore, limited proteolysis of both GmaR and MogR provides further evidence that GmaR, and not MogR, undergoes a temperature-dependent conformational change.

**Figure 6:**
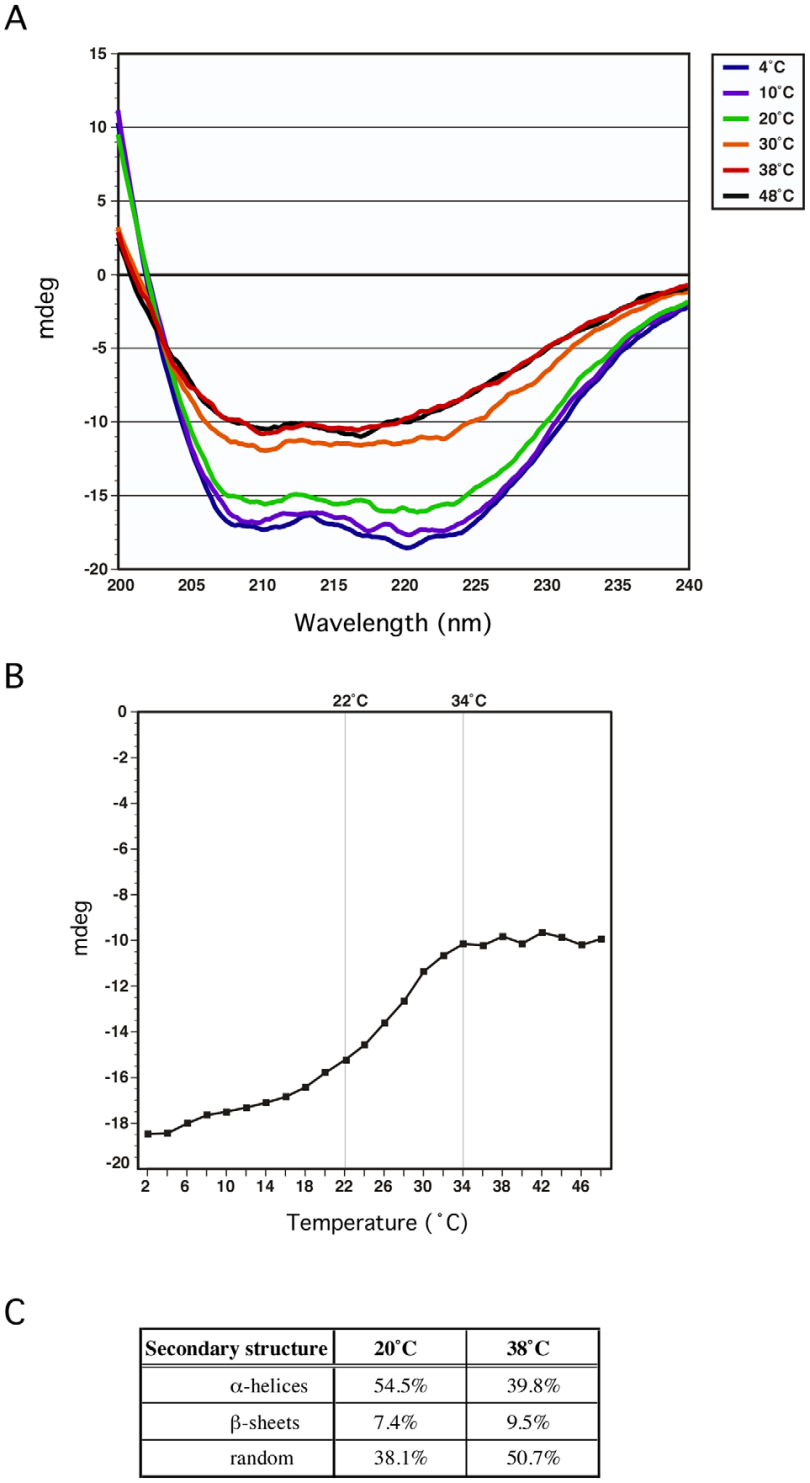
GmaR undergoes a temperature-dependent conformational change. (**A**) Circular Dichroism (CD) spectral analysis of 5 µM GmaR measured from 200 nm to 240 nm on a Jasco J-815 spectrometer at increasing temperatures (4°C, 10°C, 20°C, 30°C, 38°C and 48°C). (**B**) CD analysis of 5 µM GmaR at 220 nm over a temperature range of 2°C to 48°C with a temperature slope of 1°C/min. (**C**) Predicted secondary structure analysis of GmaR at both 20°C and 38°C estimated using the K2D2 algorithm [45].

To confirm that a temperature-dependent conformational change in GmaR occurs, we used circular dichroism (CD) spectral analysis to examine the secondary structure of GmaR. Using a wavelength scan between 200 nm and 240 nm, the secondary structure of GmaR was examined upon increasing temperature. As the temperature was raised from 4°C to 20°C, the structure of GmaR remained constant (Figure 6A). However, between 20°C and 30°C the secondary structure of GmaR drastically changed, as shown by the change in the shape of the curve at these temperatures (Figure 6A). Further analysis of structural changes at 220 nm between the temperature range of 2°C and 48°C revealed that the slope of the change was most significant between 22°C and 34°C (Figure 6B). GmaR appeared to have completed the conformational change *in vitro* at 34°C, as the secondary structure remained constant at temperatures above 34°C (Figure 6A and 6B). Additionally, the temperature-induced conformational change in GmaR was irreversible. CD spectral analysis of purified GmaR maintained at 25°C and then at 38°C indicated that GmaR did not revert back to the previous conformation when the temperature was then decreased to 25°C (Figure S5). It is not surprising that a temperature-dependent conformational change is detected prior to 37°C *in vitro*, since *in vitro* buffer conditions required for CD analysis are very different from physiological conditions within the bacterial cytoplasm. In addition, these analyses do not consider the possible impact of MogR stabilization on GmaR conformation, which may alter the temperature specificity of the conformational change in GmaR. Unfortunately, we were unable to analyze MogR by CD analysis with or without GmaR due to limitations in buffer compatibility required for both CD analysis and MogR purification. Secondary structure prediction analysis software based on homology modeling [44], identified GmaR as a highly alpha-helical protein (Figure S6). Consistent with the homology modeling results, secondary structure prediction analysis [45] based on the CD results indicates that GmaR appears to be changing from a more alpha helical protein conformation at lower temperatures (54% α-helical, 38% random) to a less structured protein conformation (39% α-helical, 50% random) at elevated temperatures (Figure 6C). This temperature-dependent change in GmaR conformation likely affects the binding interaction with MogR and destabilizes the MogR:GmaR anti-repression complex at elevated temperatures.

## Discussion

The transition from ambient temperature to physiological temperature is an important signal that bacterial pathogens sense upon entry into a mammalian host. Temperature signals can be transduced into transcriptional and translational responses necessary for adaptation to changing temperatures. In this study, we identified the temperature-responsive component that mediates temperature-dependent transcription of flagellar motility genes in *Lm*. We determined that the bifunctional flagellar anti-repressor/glycosyltransferase, GmaR, is also a protein thermometer that transduces temperature changes into a transcriptional response through its anti-repression function. We demonstrated that GmaR undergoes a temperature-dependent conformational change that affects the formation of the MogR:GmaR anti-repression complex and alters GmaR stability. Disruption of MogR:GmaR complex formation at temperatures of 37°C and above results in MogR repression of all flagellar motility genes. Therefore, GmaR functions as a thermo-sensing anti-repressor that incorporates temperature fluctuations into the transcriptional response that controls flagellar motility genes. Through this temperature-sensing mechanism, *Lm* is able to respond to physiological temperature and down-regulate flagellar motility genes once inside the host. To our knowledge, this is the first known example of an anti-repressor that transduces temperature cues into a transcriptional response required for a developmental process.

### GmaR is a protein thermometer

Thermosensors undergo temperature-dependent conformational changes that have downstream effects on transcription, translation or protein production. Most relevant to this study are the protein thermometers. To date, there have only been three other thermo-sensing proteins identified that also function as transcriptional regulators in which temperature-dependent conformational changes in either a transcriptional repressor (RheA and TlpA) or an activator (RovA) alter DNA-binding ability [21], [46], [47]. Here, we described a novel type of thermosensor, a thermo-responsive anti-repressor where a conformational change disrupts a protein-protein interaction that directly results in an altered transcriptional response.

Using limited proteolysis and Circular Dichroism (CD), our results indicated that the bifunctional anti-repressor/glycosyltransferase, GmaR, undergoes a temperature-dependent conformational change (Figures 5, S4, and 6). Secondary structure prediction analyses based on both homology and CD spectrum confirm that GmaR contains a highly alpha helical structure (Figure 6A, 6C, and S6). At temperatures above 34°C, GmaR loses some of its alpha helical structure (from 54% to 39%) (Figure 6B and 6C). CD spectral analysis also revealed that the temperature-induced conformational change in GmaR appears to be irreversible (Figure S5). CD spectral analysis of the thermo-sensing RovA activator in *Yersinia pestis* identified a similar temperature-dependent conformational change in alpha helical structure from 54% to 42% upon temperature shift from 25°C to 37°C that is reversible [21]. In the same study, temperature-dependent conformational changes were not detected in the negative controls of the *Yersinia* RovM repressor or lysozyme [21]. It is hypothesized that the temperature-dependent changes in RovA are required for *Yersinia* pathogenesis. CD spectral analysis has been used to confirm temperature-dependent conformational changes in several previously identified protein thermometers [46], [47], [48], [49], [50].

### GmaR functions as a thermo-sensing anti-repressor

We have previously shown that GmaR and MogR directly interact to form a protein-protein complex [38]. This anti-repression complex occurs at low temperatures to relieve MogR repression of flagellar motility gene transcription [38]. Using *E. coli* two-hybrid and gel mobility shift analyses, we demonstrated that the MogR:GmaR complex forms more readily at 30°C than at 37°C (Figures 2, S2 and 4), indicating that the interaction between MogR and GmaR is stronger at lower temperatures. Quantitative analysis of the MogR:GmaR interaction in the *E. coli* two-hybrid assay indicates at least a 3-fold stronger interaction between GmaR and MogR at 30°C compared to 37°C (Figure 2B). In *Campylobacter jejuni*, a similar temperature-sensitive complex between the FliA sigma factor and the FlgM anti-sigma factor was determined to control flagellum length, however the thermo-sensing component remains to be identified [51]. Our data indicates that the temperature-dependent interaction between the MogR repressor and the GmaR anti-repressor is due to temperature-dependent conformational changes in GmaR (Figure 5, S4, and 6) and not MogR (Figure 3, 5, and S4). The irreversibility of the temperature-induced conformational change in GmaR (Figure S5) suggests that once the MogR:GmaR anti-repression complex is disrupted, a new anti-repression complex with MogR can not be formed until additional GmaR is produced.

GmaR is a bifunctional protein that acts as both a glycosyltransferase and an anti-repressor [38]. Two-hybrid analysis of GmaR truncations revealed that the temperature-sensing domain of GmaR is located in the C-terminus (aa 351–637), which does not include the glycosyltransferase domain or the TPR repeat region (Figure 2 and S2). This C-terminal region of GmaR contains the MogR-binding anti-repressor domain of GmaR and has been shown to be sufficient to relieve MogR repression in *Lm* (A. Shen and D. Higgins, unpublished). Since the interaction between MogR and GmaR_351-637_ is temperature-dependent, this suggests that the thermo-responsive portion of GmaR is located in the C-terminal anti-repressor domain (Figure 2 and S2).

As temperature increases, temperature-dependent conformational changes in protein thermometers often reduce protein-protein interactions either between identical proteins (dimerization or multimerization) or between two binding protein partners. For example, a shift from low to high temperature induces conformational changes in the *S. enterica* serovar *typhi* TlpA repressor, shifting it from an active dimer to an inactive monomer [47]. Whereas DegP in *E. coli* switches from a 12–24-mer multimeric chaperone to a hexameric heat shock protease upon temperature shift [52]. Our data suggests that the temperature-dependent conformational change in GmaR (Figures 5, S4, and 6) affects the binding interaction with MogR (Figures 2, S2 and 4). The stoichiometry of the MogR:GmaR anti-repression complex is unknown and may contain multiple MogR and/or GmaR subunits. MogR binds flagellar promoter region DNA as a dimer [42], therefore the MogR:GmaR anti-repression complex could be a dimer:dimer interaction. Crystal structure studies of GmaR alone and GmaR complexed with MogR should provide insights into these questions.

GmaR is produced in a temperature-dependent manner, due to transcriptional and post-transcriptional regulation, while both MogR and DegU are constitutively expressed independent of temperature [38], [39], [40]. Therefore, temperature-dependent expression of GmaR controls temperature-dependent transcription of flagellar motility genes in *Lm*. Our data also suggests that the MogR:GmaR anti-repression complex functions to stabilize GmaR protein levels. Protein stability studies revealed that the half-life of GmaR is greatly reduced in the absence of MogR (Figure 1). GmaR is also degraded at 37°C in wild-type bacteria, a condition where the MogR:GmaR complex is disrupted due to temperature-dependent conformational changes in GmaR (Figure 1). Furthermore, GmaR not bound to MogR is unstable and susceptible to proteolysis at both 30°C and 37°C (Figure 1). In *Yersinia*, the thermo-sensing RovA activator is degraded in a temperature-dependent manner by the Lon and Clp proteases [21]. Temperature-dependent conformational changes in RovA, make RovA more susceptible to proteolysis. In contrast, GmaR is degraded in a temperature-independent manner in the absence of MogR, therefore the temperature-dependent conformational change in GmaR is important for modulating the interaction with MogR and is not required for degradation of GmaR. This result also suggests that the protease(s) responsible for GmaR degradation are constitutively expressed. In addition, unlike RovA in *Yersinia*, GmaR was not degraded in *E. coli*. Regardless of the *Lm* protease(s) responsible, degradation of GmaR is secondary to the temperature-dependent conformational change that affects MogR:GmaR complex formation and anti-repression function.

### Model for temperature-dependent control of flagellar motility gene transcription in *L. monocytogenes*

**Figure 7:**
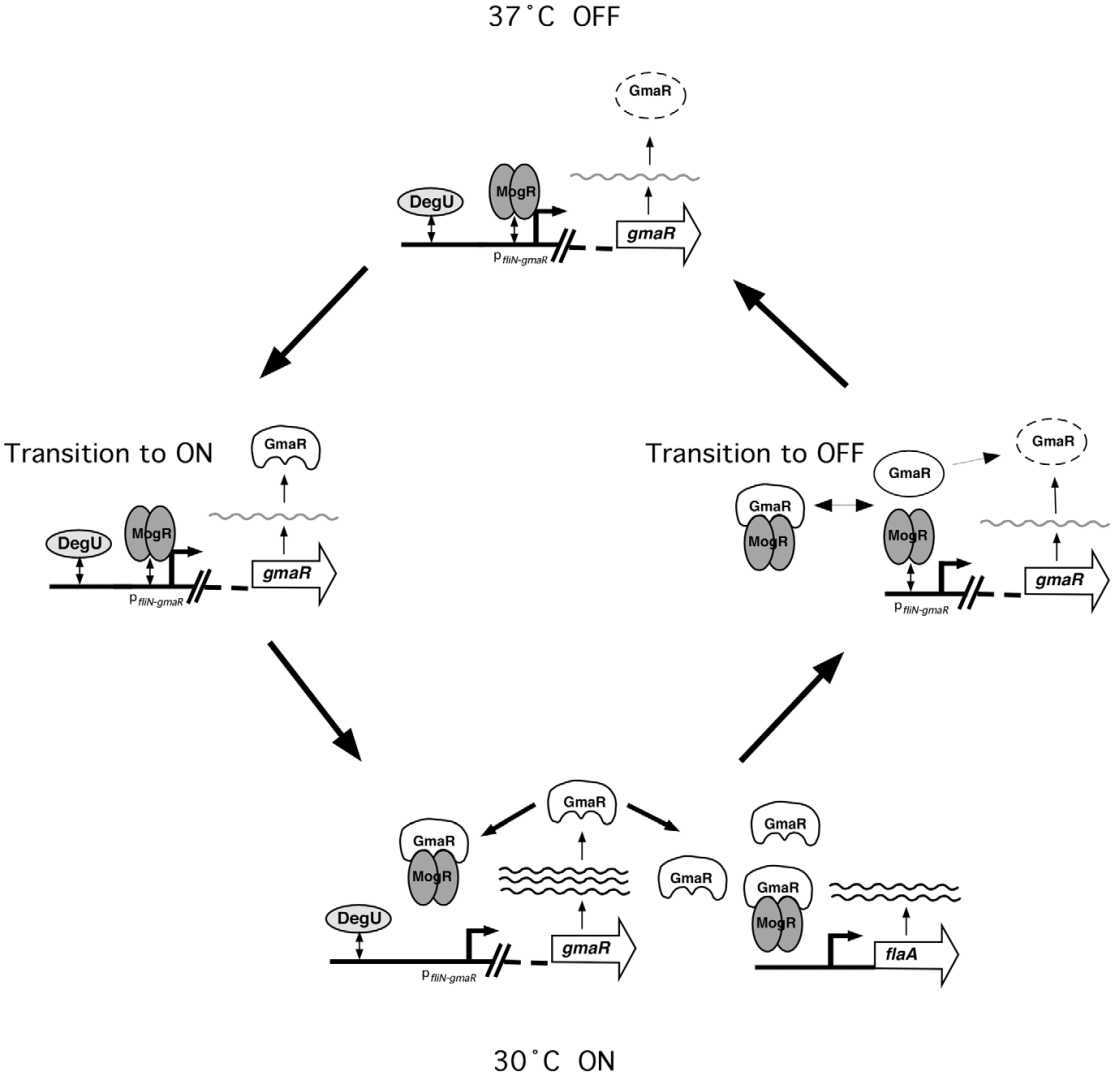
Model for the temperature-dependent regulation of GmaR expression and flagellar motility gene transcription in *Lm*. **37°C OFF**: At 37°C, when flagellar motility is OFF, the opposing activities of the MogR repressor and the DegU activator at p*_fliN-gmaR_* results in minimal *fliN-gmaR* transcripts. Any GmaR produced at 37°C is rapidly degraded and cannot interact with MogR. **Transition to ON**: As the temperature decreases below 37°C, newly synthesized GmaR produced from *fliN-gmaR* transcripts is no longer degraded and is able to interact with MogR. **30°C ON**: Once GmaR is initially translated at lower temperatures, GmaR removes MogR from the *fliN-gmaR* promoter, increasing transcription of *gmaR.* Elevated levels of GmaR results in anti-repression of all flagellar motility gene promoters, allowing flagellar motility gene transcription to occur. **Transition to OFF**: As the temperature increases, the MogR:GmaR complex is destabilized due to a temperature-dependent conformational change in GmaR. Released GmaR is degraded, while released MogR binds flagellar promoter region DNA to reinstate repression of *gmaR* and all flagellar motility genes.

Production of GmaR is the first committed step for flagellar motility in *Lm* [38]. Based on the studies presented in this report, we favor the following model for temperature-dependent regulation of GmaR expression and flagellar motility gene transcription in *Lm* (Figure 7). At 37°C and above, when flagellar motility is OFF, MogR binds and represses all flagellar motility gene promoters. The DegU response regulator can bind DNA upstream of the *fliN-gmaR* promoter and activate transcription, but due to MogR repression, transcription of *fliN-gmaR* is minimal at elevated temperatures [40]. The minimal amount of GmaR protein produced from *gmaR* transcripts at 37°C is unable to bind to MogR efficiently due to the unfavorable structural conformation of GmaR at 37°C, and is rapidly degraded. Consequently, flagellar motility gene transcription remains repressed (Figure 7, 37°C OFF). As the temperature drops below 37°C, the newly synthesized GmaR protein now produced from *gmaR* transcripts is in a favorable conformation to bind MogR (Figure 7, Transition to ON) and the MogR:GmaR anti-repression complex can now be formed (Figure 7, 30°C ON). GmaR anti-repression activity removes MogR bound to the *fliN-gmaR* promoter and permits transcriptional activation by DegU. Elevated levels of GmaR protein can then remove MogR from all flagellar motility gene promoters allowing flagellar motility gene transcription at low temperatures. During the transition from ON to OFF (30°C to 37°C), the increase in temperature induces a conformational change in GmaR that destabilizes the MogR:GmaR complex, releasing MogR to bind flagellar promoter region DNA and reinstate repression of *gmaR* transcription. GmaR protein that is released from the MogR:GmaR complex can no longer bind MogR and is degraded (Figure 7, Transition to OFF). New GmaR protein must be produced to re-establish transcription of flagellar motility genes at low temperatures. It should be noted that experiments presented in this report were performed under *in vitro* conditions and that our working model is open for future amendment. While the *in vitro* experiments provide strong evidence that GmaR undergoes a temperature-dependent conformational change that disrupts the MogR:GmaR complex, we do not provide direct evidence of this mechanism occurring in *L. monocytogenes*.

*Lm* is a facultative intracellular pathogen that infects host cells, typically at physiological temperatures of 37°C and above. In response to temperature, *Lm* reciprocally regulates its virulence and flagellar motility genes. An RNA thermometer was previously identified in *Lm* that controls translation of the critical virulence regulator PrfA [12]. We have now identified a protein thermometer, GmaR that controls temperature-dependent transcription of flagellar motility genes in *Lm*. The combination of these two thermosensors (RNA and protein) allows for reciprocal regulation of virulence genes and flagellar motility genes in *Lm*. At physiological temperatures (37°C and above) when the RNA thermometer structure within *prfA* transcripts melts to allow translation of PrfA and up-regulation of virulence genes, the thermo-sensing flagellar anti-repressor GmaR, releases the MogR repressor to reinstate repression of flagellar motility genes. Down-regulation of flagellar motility during host infection is an important aspect of pathogenesis since flagella are recognized by the host immune system, are unnecessary inside the host cell cytosol where actin-based motility occurs, and are energetically unfavorable. Likewise, when *Lm* is in the extracellular environment at temperatures below 37°C when flagellar motility is required for chemotaxis and biofilm formation, the RNA thermometer in *prfA* prevents transcription of virulence genes. It is not surprising that as a facultative intracellular pathogen able to inhabit a wide range of environments, *Lm* uses thermosensors tuned to environmental temperatures. In fact, it is very likely that there are additional thermo-sensing elements in *Lm* that are yet to be identified. In this study, we have identified the first protein thermometer required for temperature-dependent transcription of flagellar motility genes and demonstrated how temperature signals are transduced through a protein:protein (anti-repressor:repressor) interaction to generate a transcriptional response.

## Materials and Methods

### Bacterial strains and growth media

*Escherichia coli* (*Ec*) and *Listeria monocytogenes* (*Lm*) strains used in this study are listed in Supporting Table S1. Specific details for construction of bacterial strains are located in Supporting Materials and Methods in Text S1. Primers used in this study are listed in Supporting Table S2. *Ec* strains were grown in Luria-Bertani (LB) medium except where noted. All *Lm* strains are derived from wild-type strain EGDe and were grown in Brain Heart Infusion (BHI) broth. Antibiotics were used at the following concentrations: chloramphenicol at 25 µg/mL for selection of pAC derivatives in *Ec*; 100 µg/mL carbenicillin for pBR derivatives in *Ec*; 30 µg/mL kanamycin for the FW102 O_L_2-62 reporter strain and pET derivatives in *Ec*. All plasmid constructs were confirmed by automated sequencing. Plasmids were isolated from XL1-Blue or DH5α prior to transformation into the assay strains.

### Purification of GmaR-His_6_

Ten grams of frozen cell pellet (fermentor run details are located in the Supporting Materials and Methods in Text S1) were thawed and homogenized in a total volume of 45 mL of cold column binding buffer (20 mM NaH_2_PO_4_, 0.5 M NaCl, 20 mM Imidazole, pH 7.4), in a 50 mL conical tube in an ice-water bath. A pre-chilled sonication probe was used at 95% amplitude to lyse the cells in an ice-water bath using three rounds of three repeats of 10 sec sonication cycles (a total of 30 sec of sonication per round) with a 10 sec rest on ice between each repeat. Cell debris was pelleted in the conical tube (23,400× *g* at 4°C for 20 min). The culture supernatant was removed and re-centrifuged in a 50 mL conical tube before being filtered through a 0.8/0.22 µm VacuCap 90 PF filter (Pall Life Sciences, Ann Arbor, MI). Filtered lysate was loaded onto a pre-packed Ni column (HisTrap HP 5 mL column, GE Healthcare, Piscataway, NJ) at 2 mL/min using the Biologic Chromatography system (Bio-Rad Laboratories, Hercules, CA). The column was washed with 6 column volumes of ice cold binding buffer at 2 mL/min to remove weakly bound proteins. The recombinant protein was then eluted at 2 mL/min using a gradient of increasing elution buffer (50 mM NaH_2_PO_4_, 0.3 M NaCl, 500 mM Imidazole, pH 7.4). Fractions were collected and analyzed by SDS-PAGE and Western blot (using anti-histidine tag antibody) to identify those containing GmaR-His_6_. Positive fractions (elution at 22% elution buffer, 78% binding buffer) were pooled, passed through a 0.45 µm filter, and loaded onto a HiLoad 26/60 Superdex 200 prep grade gel filtration column (GE Healthcare Life Sciences) pre-equilibrated with running buffer (0.5 M NaCl, 20 mM NaH_2_PO_4_, pH 7.5). Fractions were collected and analyzed by SDS-PAGE and Coomassie stain. Positive fractions were pooled and dialyzed (10% glycerol, 10 mM NaCl, 20 mM NaH_2_PO_4_, pH 7.0), concentrated to a 5 mL volume and frozen at −80°C. Final protein concentration was determined by Bradford analysis using a BSA standard to be 14 mg/mL.

### Purification of MogR-His_6_

Ni^2+^-affinity purification of His_6_-tagged MogR was performed as previously described [39].

### Translation inhibition assay

A single colony of either WT or Δ*mogR* strain was inoculated into 30 mL of BHI broth in a 250 mL flask and grown 16–18 h shaking at 30°C (1° culture). The next day, 3 mL of the 1° culture was inoculated into 150 mL of BHI broth in a 2 L flask and left standing at RT for 16–18 h (2° culture). On day 3, the 2° culture (OD_600_∼1.0) was diluted to OD_600_ = 0.4 with BHI broth and split into 4 flasks for each strain. For the first time point (T = 0), 7 mL of culture was removed. At time zero, 8 µg/mL of tetracycline was added to two of the four flasks, and then one treated and untreated flask for each strain was placed standing at both 37°C and 30°C. For each time point (0, 1, 2, 3, 4, 5, 6, 7, 8 h), 0.9 mL was used to determine the OD_600_; 0.1 mL was diluted, spread on BHI plates, and incubated 16–18 h to determine cfu/mL (at time point 0, 4, and 8 h only); and 6 mL was pelleted, resuspended in 100 µl of TE/lysozyme (10 mM Tris-HCL [pH 8.0], 1 mM EDTA, 3 mg/mL lysozyme) and incubated at 37°C for 1 h. After the 1 h incubation at 37°C, an equal volume of 2X SDS loading buffer was added. Samples were boiled for 5 min at 95°C and then centrifuged for 1 min at 16,000× *g*. 30 µL of the boiled sample was loaded onto a 6% SDS-PAGE gel for analysis of GmaR or a 12% SDS-PAGE gel for analysis of DegU.

### Quantitative Western blot analysis

Western blot analysis was performed using a polyclonal antibody specific for either GmaR or DegU and a goat anti-rabbit horseradish peroxidase-conjugated secondary antibody (BioRad) and the Western Lightning (Perkin Elmer) ECL detection method. Western blots of WT and Δ*mogR* at both temperatures were exposed together on the same film for each experiment. ImageJ software (NIH) was used to perform densitometry quantification of three independent experiments for the translation inhibition assay. GmaR protein present at T = 0 in each strain was set to 100% and used to calculate % degradation. The average and standard deviation of the mean of three independent experiments was graphed.

### Gel mobility shift analysis

Gel mobility shift analysis was performed as previously described [39], [40].

### Two-Hybrid β-galactosidase assays

FW102 O_L_2-62 reporter strain cells were co-transformed with the indicated pAC- and pBR-derived plasmids. Three colonies for each co-transformant were inoculated into 4 mL of LB containing 100 µg/mL of carbenicillin, 30 µg/mL of kanamycin, and 25 µg/ml of chloramphenicol, the cultures were split into 2×2 mL and placed at both 30°C and 37°C for 16–18 h on a roller drum. The next day, 20 µL of each culture was placed into 2 mL of LB containing 100 µg/mL of carbenicillin, 30 µg/mL of kanamycin, and 25 µg/mL of chloramphenicol and 10 µM IPTG that had been pre-equilibrated to either 30°C or 37°C. Cultures were grown 2–3 h until OD_600_ ∼0.3. β-galactosidase assays were performed and Miller units were calculated as described [53] by using microtiter plates and a microtiter plate reader. Values represent the means from three independent measurements obtained on the same day and their standard deviations. Assays were conducted three times in triplicate on separate days with similar results.

### Chymotrypsin analysis

Purified GmaR-His_6_ or MogR-His_6_ was pre-incubated at either RT or 37°C in digestion buffer (10 mM Tris, pH 7.5) for 5 min. Chymotrypsin was then added to the purified protein at a concentration of 1∶5000. A 10 µL volume containing 5 µg of purified protein was removed at 0, 1, 2, 5, 10, 20, 30 and 60 min and immediately mixed with 2X loading buffer to stop digestion. The entire sample (20 µL) for each time point was run on a 10% SDS-PAGE gel and stained with Coomassie for analysis.

### Circular dichroism (CD) spectral analysis

Purified GmaR-His_6_ (186 µM stock in 10% glycerol, 10 mM NaCl, 20 mM NaH_2_PO_4_, pH 7.0), was diluted to 5 µM in CD buffer (10 mM NaH_2_PO_4_, pH 7.0). A Jasco J-815 spectrometer with a thermo-stated cell holder was used for CD spectroscopy. A temperature scan from 2°C to 48°C was run, with a ramp rate of 1°C/min with a λ scan from 190–250 nm at every 2°C (reported in Figure 6A is the spectrum for 4°C, 10°C, 20°C, 30°C, 38°C, and 48°C only). Each spectrum was the result of five successive spectra that were normalized against the CD buffer run at the same temperature. In Figure 6B, the entire temperature range (2°C–48°C) is reported at a single wavelength of 220 nm. Secondary structure prediction at 20°C and 38°C was estimated by using the K2D2 algorithm [45].

## Supporting Information

Figure S1 **The translational inhibitor tetracycline is bacteriostatic at 8 µg/mL.** (**A**) Growth analysis of wild-type (WT) and Δ*mogR* (ΔM) bacteria grown with or without 8 µg/mL tetracycline. *Lm* were grown 16-18 h at RT without shaking. Cultures were diluted to an OD_600_ = 0.4, split into four samples, treated with or without tetracycline and then shifted to either 30°C or 37°C. Cultures were then grown without shaking for an additional 8 h. The OD_600_ was measured at each time point following temperature shift. Tetracycline was added to the cultures labeled ++. Samples were also collected at each time point for determination of cfu/mL (panel B) and GmaR protein analysis via Western Blot (Figure 1). (**B**) Determination of cfu/mL of WT and ΔM bacteria. Culture samples taken as described in panel A (0, 4, and 8 h) were diluted, plated on BHI agar and incubated 16-18 h at 37°C. Bacterial colonies were counted and cfu/mL for each sample determined. Data represent one of three independent experiments with similar results.
(PDF)

Figure S2 **β-galactosidase activities and fusion protein levels from** ***E. coli*** **two-hybrid analysis.** (**A**) *E. coli* two-hybrid analysis of the MogR:GmaR interaction. Black bars represent the β-galactosidase activity in Miller units of the two interacting protein fusions at the temperature indicated below the bar. Grey bars represent the background activity of the α-NTD negative control for each fusion at the same temperature. Data represent the means and standard deviations of three independent experiments performed on the same day. Assays were performed on three separate days with similar results. (**B**) Protein levels of MogR and GmaR fusions from assays performed in Figure 2B and panel A. Twenty microliters of cell lysates directly from the assay plates were analyzed by SDS-PAGE and Western blot. An anti-λcI or anti-αNTD antibody was used for detection of fusion proteins. The fusion proteins expressed in *E. coli* are: Lane 1: λcI-GmaR and αNTD-MogR_1-162_, Lane 2: λcI-GmaR and empty αNTD, Lane 3: λcI-MogR and αNTD-GmaR, Lane 4: λcI-MogR and αNTD-GmaR_165-637_, Lane 5: λcI-MogR and empty αNTD, Lane 6: Empty λcI and αNTD-MogR_1-162_, Lane 7: λcI-GmaR_165-637_ and αNTD-MogR_1-162_, Lane 8: λcI-GmaR_165-637_ and empty αNTD. The native αNTD of *E. coli* RNA polymerase was also detected by the anti-αNTD antibody (*).
(PDF)

Figure S3 **DNA sequence of the** ***fliN-gmaR*** **and** ***flaA*** **promoter regions.** The transcriptional start sites were previously mapped by primer extension and are marked with +1 and underlined. The –35 and –10 promoter sequences are indicated and underlined. The MogR binding sites (5′-TTTTWWNWWAAAA-3′) as predicted by crystal structure analysis [1] are shaded in grey.
(PDF)

Figure S4 **Limited proteolysis of GmaR and MogR with trypsin indicates a temperature-dependent conformational change in GmaR, but not MogR.** Purified GmaR-His_6_ or MogR-His_6_ was incubated with trypsin (10,000∶1) for 60 min at either RT or 37°C. Reactions were stopped at the sample times indicated by removing 10 µg of protein and mixing with 2X loading buffer. Samples were run on an SDS-PAGE gel and stained with Coomassie stain.
(PDF)

Figure S5 **Conformational changes in GmaR are irreversible.** Circular Dichroism (CD) spectral analysis of 5 µM GmaR measured from 200 nm to 240 nm on a Jasco J-815 spectrometer at 25°C, 38°C and then again at 25°C.
(PDF)

Figure S6 **Predicted secondary structure of GmaR.** (**A**) GmaR amino acid sequence color-coded by secondary structure prediction as determined by homology modeling using the Phyre database [2]. Green denotes random coil, Red denotes α-helix, Blue denotes β-sheet. The dashed underline marks the glycosyltransferase domain. The solid underline marks the TPR region. (**B**) Predicted secondary structure analysis of GmaR based on data presented in A.
(PDF)

Table S1 ***Listeria monocytogenes*** **and** ***Escherichia coli*** **strains.**
(PDF)

Table S2 **Oligonucleotides used in this study.**
(PDF)

Text S1 **Includes additional experimental procedures used for bacterial strain construction, fermentor run, and trypsin analysis; bibliography of references cited in the Supporting Information materials.**
(PDF)
