# Supplementary material for: A Protein Thermometer Controls Temperature-Dependent Transcription of Flagellar Motility Genes in Listeria monocytogenes
Source: PLoS Pathog. 2011 Aug 4;7(8):e1002153. doi: 10.1371/journal.ppat.1002153 (PMC3150276; doi:10.1371/journal.ppat.1002153)
Supplement: Figure S1 — The translational inhibitor tetracycline is bacteriostatic at 8 µg/mL. (A) Growth analysis of wild-type (WT) and ΔmogR (ΔM) bacteria grown with or without 8 µg/mL tetracycline. Lm were grown 16-18 h at RT without shaking. Cultures were diluted to an OD600 = 0.4, split into four samples, treated with or without tetracycline and then shifted to either 30°C or 37°C. Cultures were then grown without shaking for an additional 8 h. The OD600 was measured at each time point following temperature shift. Tetracycline was added to the cultures labeled ++. Samples were also collected at each time point for determination of cfu/mL (panel B) and GmaR protein analysis via Western Blot (Figure 1). (B) Determination of cfu/mL of WT and ΔM bacteria. Culture samples taken as described in panel A (0, 4, and 8 h) were diluted, plated on BHI agar and incubated 16-18 h at 37°C. Bacterial colonies were counted and cfu/mL for each sample determined. Data represent one of three independent experiments with similar results. (PDF) [file ppat.1002153.s001.pdf]

Kamp_FigS1

A

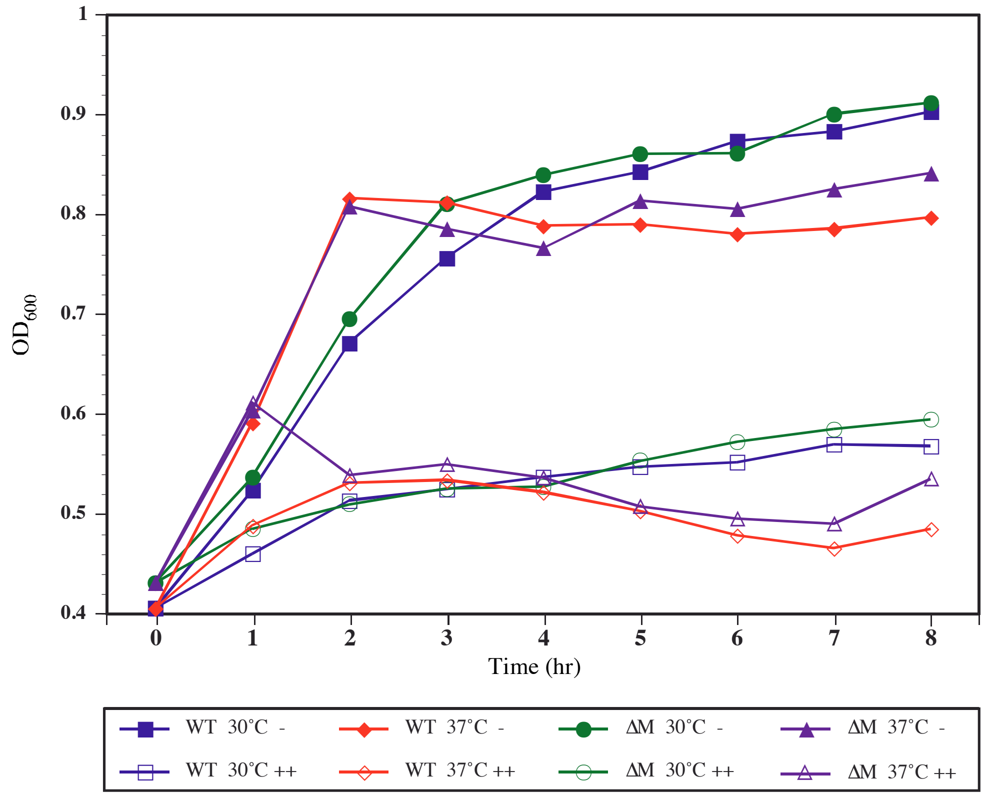

B

	WT 30°C		WT 37°C		ΔM 30°C		ΔM 37°C	
Time (hr)	**-**	**++**	**-**	**++**	**-**	**++**	**-**	**++**
0	2.80×10^8	2.80×10^8	2.80×10^8	2.80×10^8	2.70×10^8	2.70×10^8	2.70×10^8	2.70×10^8
4	1.04×10^9	4.00×10^8	1.06×10^9	3.90×10^8	4.30×10^8	3.80×10^8	5.10×10^8	2.49×10^8
8	1.37×10^9	3.50×10^8	1.07×10^9	2.32×10^8	2.99×10^9	2.90×10^8	1.65×10^9	1.87×10^8
