# Supplementary figures and images for: A Protein Thermometer Controls Temperature-Dependent Transcription of Flagellar Motility Genes in Listeria monocytogenes
Source: PLoS Pathog. 2011 Aug 4;7(8):e1002153. doi: 10.1371/journal.ppat.1002153 (PMC3150276; doi:10.1371/journal.ppat.1002153)

A

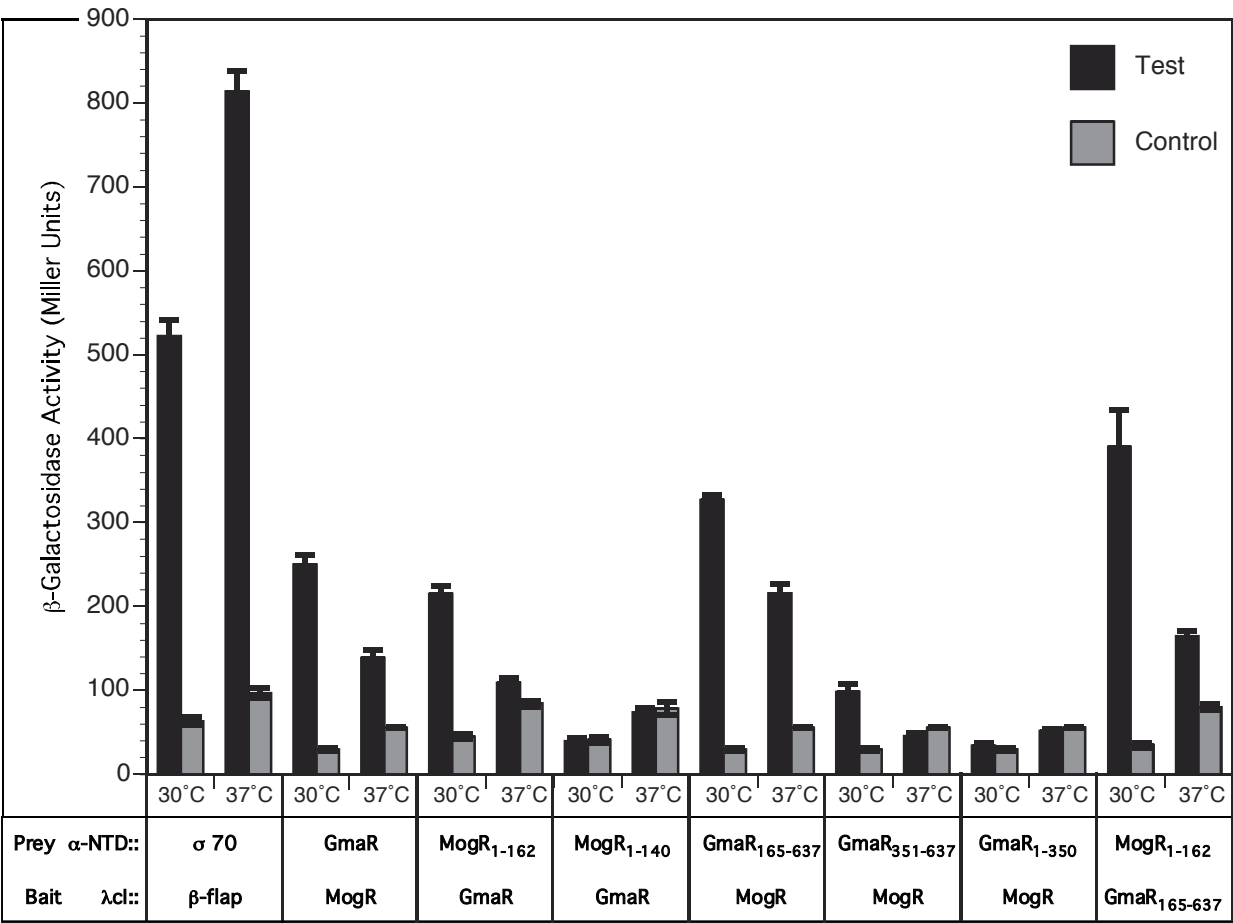

B

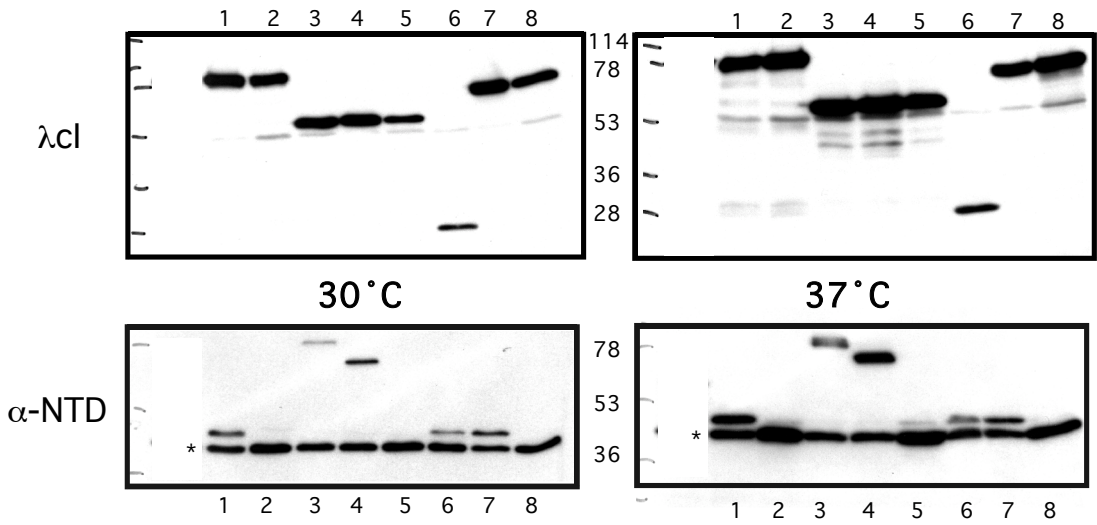

Supplement: Figure S2 — β-galactosidase activities and fusion protein levels from E. coli two-hybrid analysis. (A) E. coli two-hybrid analysis of the MogR:GmaR interaction. Black bars represent the β-galactosidase activity in Miller units of the two interacting protein fusions at the temperature indicated below the bar. Grey bars represent the background activity of the α-NTD negative control for each fusion at the same temperature. Data represent the means and standard deviations of three independent experiments performed on the same day. Assays were performed on three separate days with similar results. (B) Protein levels of MogR and GmaR fusions from assays performed in Figure 2B and panel A. Twenty microliters of cell lysates directly from the assay plates were analyzed by SDS-PAGE and Western blot. An anti-λcI or anti-αNTD antibody was used for detection of fusion proteins. The fusion proteins expressed in E. coli are: Lane 1: λcI-GmaR and αNTD-MogR1-162, Lane 2: λcI-GmaR and empty αNTD, Lane 3: λcI-MogR and αNTD-GmaR, Lane 4: λcI-MogR and αNTD-GmaR165-637, Lane 5: λcI-MogR and empty αNTD, Lane 6: Empty λcI and αNTD-MogR1-162, Lane 7: λcI-GmaR165-637 and αNTD-MogR1-162, Lane 8: λcI-GmaR165-637 and empty αNTD. The native αNTD of E. coli RNA polymerase was also detected by the anti-αNTD antibody (*). (PDF) [file ppat.1002153.s002.pdf]

# Kamp\_FigS4

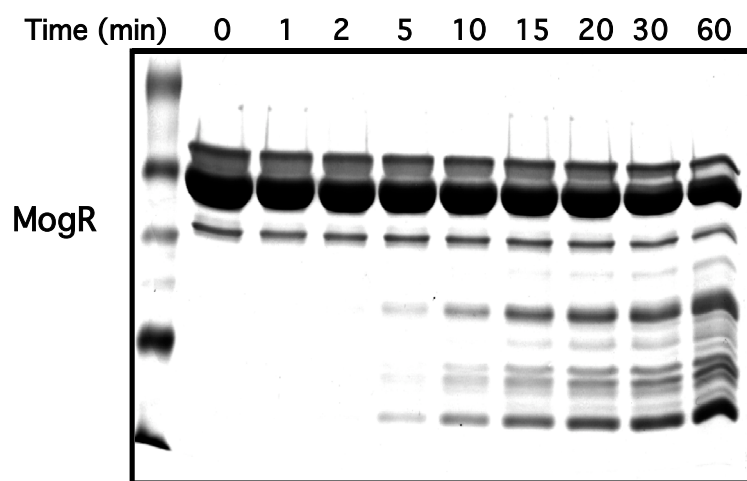

RT

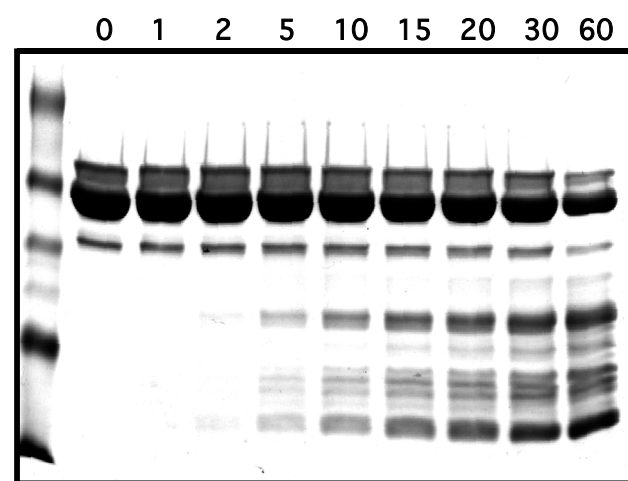

37°C

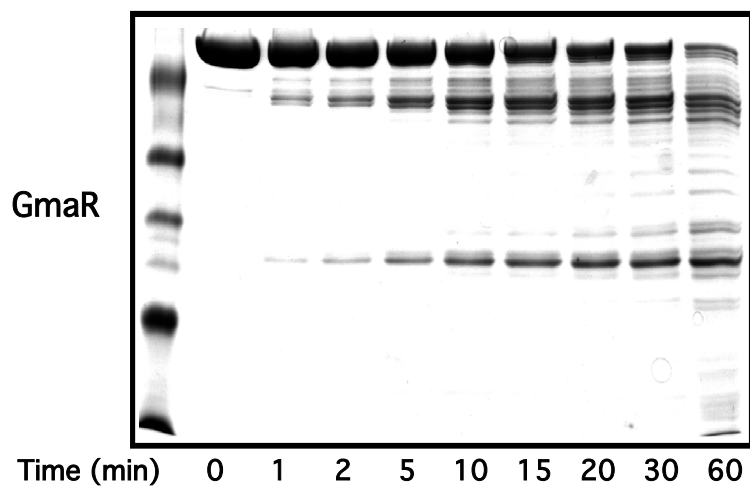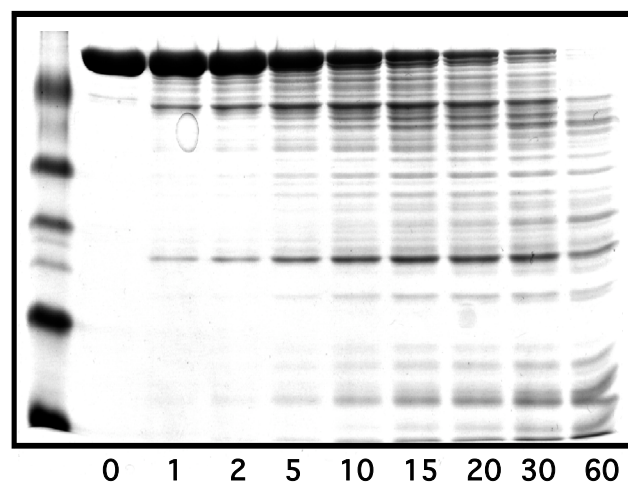

Supplement: Figure S4 — Limited proteolysis of GmaR and MogR with trypsin indicates a temperature-dependent conformational change in GmaR, but not MogR. Purified GmaR-His6 or MogR-His6 was incubated with trypsin (10,000∶1) for 60 min at either RT or 37°C. Reactions were stopped at the sample times indicated by removing 10 µg of protein and mixing with 2X loading buffer. Samples were run on an SDS-PAGE gel and stained with Coomassie stain. (PDF) [file ppat.1002153.s004.pdf]

# Kamp\_FigS5

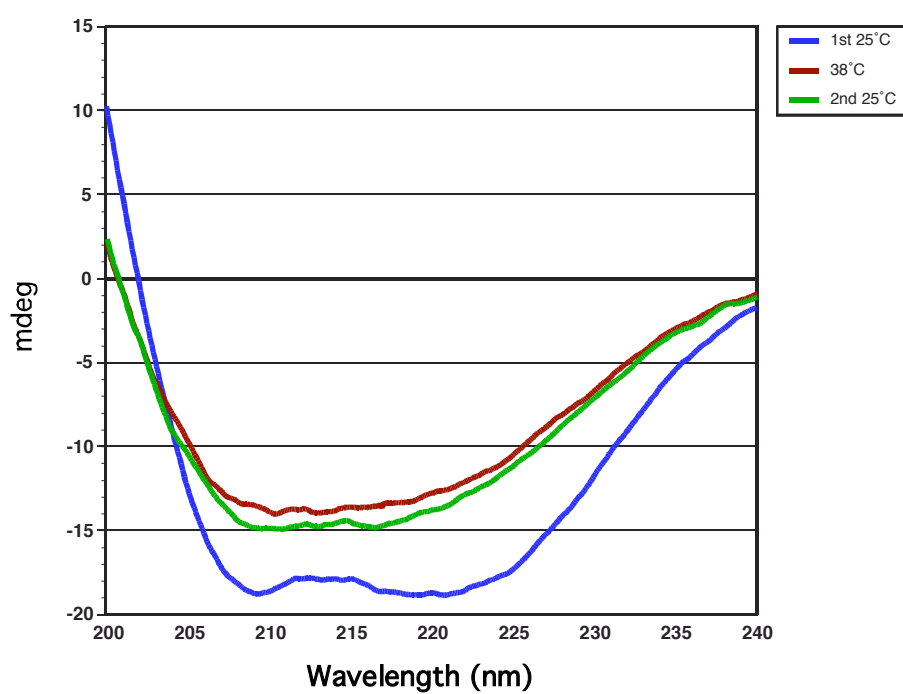

Supplement: Figure S5 — Conformational changes in GmaR are irreversible. Circular Dichroism (CD) spectral analysis of 5 µM GmaR measured from 200 nm to 240 nm on a Jasco J-815 spectrometer at 25°C, 38°C and then again at 25°C. (PDF) [file ppat.1002153.s005.pdf]

# Kamp\_FigS6

A

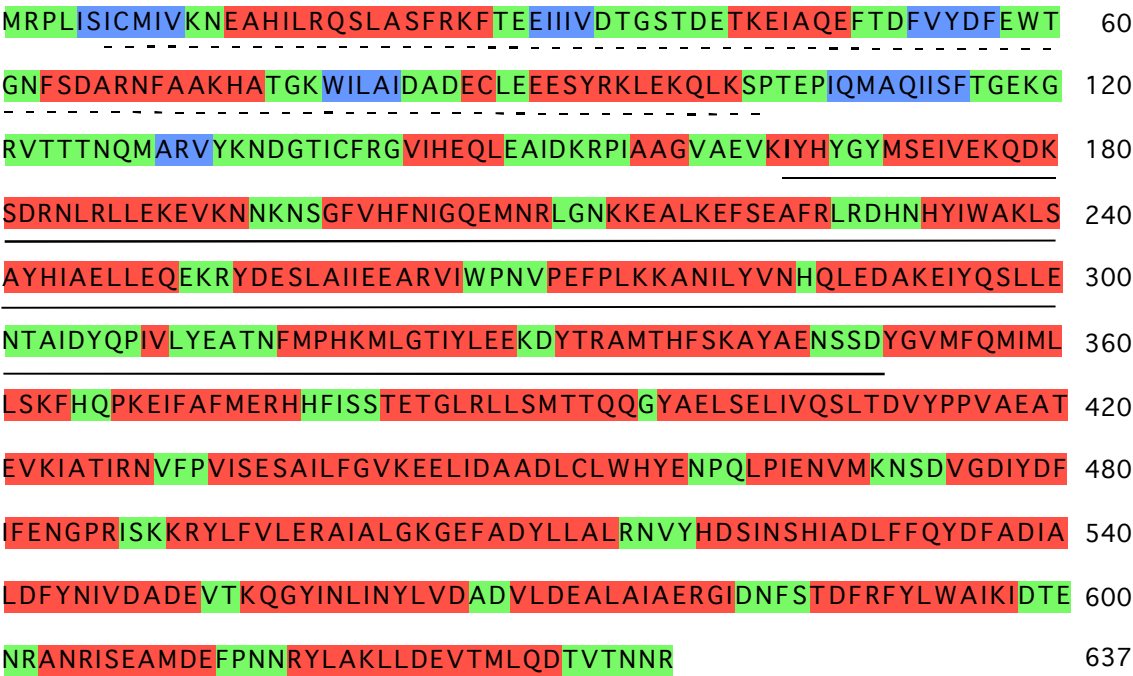

B

| Secondary structure |       |
|---------------------|-------|
| $\alpha$ -helices   | 69.1% |
| $\beta$ -sheets     | 5.3%  |
| random              | 25.6% |

Supplement: Figure S6 — Predicted secondary structure of GmaR. (A) GmaR amino acid sequence color-coded by secondary structure prediction as determined by homology modeling using the Phyre database [2]. Green denotes random coil, Red denotes α-helix, Blue denotes β-sheet. The dashed underline marks the glycosyltransferase domain. The solid underline marks the TPR region. (B) Predicted secondary structure analysis of GmaR based on data presented in A. (PDF) [file ppat.1002153.s006.pdf]
