# Supplementary material for: A Protein Thermometer Controls Temperature-Dependent Transcription of Flagellar Motility Genes in Listeria monocytogenes
Source: PLoS Pathog. 2011 Aug 4;7(8):e1002153. doi: 10.1371/journal.ppat.1002153 (PMC3150276; doi:10.1371/journal.ppat.1002153)
Supplement: Figure S3 — DNA sequence of the fliN-gmaR and flaA promoter regions. The transcriptional start sites were previously mapped by primer extension and are marked with +1 and underlined. The –35 and –10 promoter sequences are indicated and underlined. The MogR binding sites (5′-TTTTWWNWWAAAA-3′) as predicted by crystal structure analysis [1] are shaded in grey. (PDF) [file ppat.1002153.s003.pdf]

Kamp_FigS3

-35 -10 +1
TGTTTTTTTATTTTTACACACAATTTCA**TTTT**TTAAA**AAAA**GTATAGATTTTTTCAGGATTTGGTGTAAAATTTTATATTG**C** - 95 bp - **ATG**

p*fliN-gmaR*

-124
CTTCTTTA**TTTT**ATTTT**AAAA**AATAAATTTATAAAAACATAAAAACGTTGATATAAAGCCGATATTTCGTTT

-68 -35 -10 +1
TTTTATAAATAAAAACAACTTATTTTTACA**TTTT**TAATA**AAAA**ACATTTGA**TTTT**TTAAA**AAAA**TGAAGATATAATAAAGCAT**A** - 48 bp **-** **ATG**

p*flaA*
