# Supplementary material for: A Protein Thermometer Controls Temperature-Dependent Transcription of Flagellar Motility Genes in Listeria monocytogenes
Source: PLoS Pathog. 2011 Aug 4;7(8):e1002153. doi: 10.1371/journal.ppat.1002153 (PMC3150276; doi:10.1371/journal.ppat.1002153)
Supplement: Table S1 — Listeria monocytogenes and Escherichia coli strains. (PDF) [file ppat.1002153.s007.pdf]

Table S1. ***Listeria monocytogenes*** **and** ***Escherichia coli*** **strains**

Listeria monocytogenes

Strain	Genotype and relevant features	Strain Designation	Reference
DH-L478	Wild-type *Lm* strain EGDe	wild-type	M. Loessner
DH-L1156	*mogR* in-frame deletion in EGDe	Δ*mogR*	[3]

Escherichia coli

Strain	Genotype and relevant features	Reference
DH-E387	BL21star(DE3)	Invitrogen
DH-E182	XL1-Blue {F' *proAB* $lacI^q$ Δ(*lacZ*)M15 Tn10} *recA1 endA1 gyrA96 thi-1 hsdR17 supE relA1 lac*	Stratagene
DH-E1335	pET29b-*mogR* in BL21(DE3)	[4]
DH-E1951	pBRα in DH5α	[5]
DH-E1952	pBRα-σ^{70} D581G in DH5α	[6]
DH-E1953	pAC-λCI in DH5α	[5]
DH-E1954	pAC-λCI-β-flap (831-1057) in DH5α	[7]
DH-E1979	pBRα-*gmaR* in XL1-Blue	This study
DH-E1981	pAC-λCI-*mogR* in XL1-Blue	This study
DH-E1982	pBRα-$mogR_{1\text{-}162}$ in XL1-Blue	This study
DH-E1983	pBRα-$mogR_{1\text{-}140}$ in XL1-Blue	This study
DH-E1984	pAC-λCI-*gmaR* in XL1-Blue	This study
DH-E1985	pBRα-$gmaR_{165\text{-}637}$ in XL1-Blue	This study
DH-E1986	pAC-λCI-$gmaR_{165\text{-}637}$ in XL1-Blue	This study
DH-E1987	pBRα-$gmaR_{351\text{-}637}$ in XL1-Blue	This study
DH-E1988	pBRα-$gmaR_{1\text{-}350}$ in XL1-Blue	This study
DH-E1989	pET29b-*gmaR* in XL1-Blue	This study
DH-E1991	pET29b-*gmaR* in BL21star(DE3)	This study
FW102 O_L2-62	FW102 harboring an F'Kan bearing test promoter p*lac*O_L2-62 linked to *lacZ*	[8]
