# Supplementary material for: A Protein Thermometer Controls Temperature-Dependent Transcription of Flagellar Motility Genes in Listeria monocytogenes
Source: PLoS Pathog. 2011 Aug 4;7(8):e1002153. doi: 10.1371/journal.ppat.1002153 (PMC3150276; doi:10.1371/journal.ppat.1002153)
Supplement: Table S2 — Oligonucleotides used in this study. (PDF) [file ppat.1002153.s008.pdf]

Table S2. Oligonucleotides used in this study

Number	Sequence	Site[a]
786	GGAATTCCATATGCGGCCGTTAATTTCGATTTG	*NdeI*
787	CCACTCGAGTCGATTGTTTGTAACAGTGTC	*XhoI*
788	GAAGGCGGCCGCAATGCCTAAATCAGAAATAAG	*NotI*
789	GAAGGGATCCTTACATTTGTTTATAATTTTC	*BamHI*
790	GAAGGGATCCTTATTCAGCTGGCACAACTTTGCC	*BamHI*
791	GAAGGGATCCTTATCTCCCAGGCTTGTTTTTCG	*BamHI*
792	GAAGGCGGCCGCAATGCGGCCGTTAATTTCGATTTG	*NotI*
793	GAAGGGATCCCTATCGATTGTTTGTAACAG	*BamHI*
794	GAAGGCGGCCGCAATGAAAATTTACCATTATGGCTATATGTCG	*NotI*
795	GAAGGGATCCCTAATCGGAACTGTTTTCCGCAT	*BamHI*
796	GAAGGCGGCCGCAATGTACGGTGTGATGTTCCA	*NotI*

a. The indicated restriction endonuclease site is underlined within the oligonucleotide sequence
