# Supplementary material for: A Protein Thermometer Controls Temperature-Dependent Transcription of Flagellar Motility Genes in Listeria monocytogenes
Source: PLoS Pathog. 2011 Aug 4;7(8):e1002153. doi: 10.1371/journal.ppat.1002153 (PMC3150276; doi:10.1371/journal.ppat.1002153)
Supplement: Text S1 — Includes additional experimental procedures used for bacterial strain construction, fermentor run, and trypsin analysis; bibliography of references cited in the Supporting Information materials. (PDF) [file ppat.1002153.s009.pdf]

Supporting Materials and Methods

Strain Construction

To clone C-terminally His_6-tagged GmaR, primer pair #786 and #787 were used with EGDe genomic DNA to amplify the *gmaR* coding sequence. The resulting PCR product was digested with *NdeI* and *XhoI* and ligated to pET29b digested with the same enzymes. The ligations were transformed into XL1-Blue, generating strain DH-E1989. The resulting plasmid pET29b-*gmaR* was transformed into BL21star(DE3), yielding strain DH-E1991.

To clone the MogR two-hybrid fusion proteins, the 5' primer #788 and the 3' primers #789, #790 or #791 were used with EGDe genomic DNA to amplify full-length MogR, $MogR_{1-162}$, and $MogR_{1-140}$ respectively. To clone the GmaR two-hybrid fusion proteins, the 5' primer #792 and the 3' primers #793 or #795 were used with EGDe genomic DNA to amplify full-length GmaR or $GmaR_{1-350}$, respectively. Primer pair #794 and #793 was used for $GmaR_{165-637}$ and primer pair #796 and #793 was used for $GmaR_{351-637}$. The resulting PCR products were digested with *NotI* and *BamHI* and ligated into both pAC-λCI and pBRα and transformed into XL1-Blue.

Fermentor Run

One liter of EPCM1 (Current Protocols in Protein Science, 1995, 5.3.1-5.3.18) containing 30 μg/mL of kanamycin was inoculated with multiple colonies of strain DH-E1991 (starter culture) and incubated shaking 16-18 h at 37˚C (final OD_{600}= 5.7). The next day, the entire volume (1 L) of starter culture was added to 4 L of pre-warmed EPCM1 containing 30 μg/mL of kanamycin in a 7 L fermentor (Applikon Biotechnology, Foster City CA). The starting OD_{600} was ~1.0. The culture was grown at 37˚C, pH 7.0, with mixing and addition of air and oxygen to maintain an excess of oxygen until an OD_{600} of 5.0 was reached (~4 h). Protein expression was induced by lowering the temperature to 30˚C before adding IPTG (0.8 mM). After 1.5 h of induction (OD_{600}=7.2), the culture was harvested by centrifugation (20 min at 6,620 x *g*, 4ºC). Culture supernatant was discarded and cell pellets frozen at -80ºC until purification. One fermentation run yielded 60 g of frozen wet-cell pellet.

Trypsin analysis

Purified GmaR-His_6 or MogR-His_6 was pre-incubated at either RT or 37˚C in digestion buffer (10 mM Tris pH 7.5) for 5 min. Trypsin was then added to the purified protein at a concentration of 1:10,000. A 10 μL volume containing 10 μg of purified protein was removed at 0, 2, 5, 10, 15, 20, 30 and 60 min and immediately mixed with 2X loading buffer to stop digestion. The entire sample (20 μL) for each time point was run on a 10% SDS-PAGE gel and stained with Coomassie for analysis.

Supporting Information References

1. Shen A, Higgins DE, Panne D (2009) Recognition of AT-rich DNA binding sites by the MogR repressor. Structure 17: 769-777.
2. Kelley LA, Sternberg MJ (2009) Protein structure prediction on the Web: a case study using the Phyre server. Nat Protoc 4: 363-371.
3. Gründling A, Burrack LS, Bouwer HG, Higgins DE (2004) *Listeria monocytogenes* regulates flagellar motility gene expression through MogR, a transcriptional repressor required for virulence. Proc Natl Acad Sci U S A 101: 12318-12323.
4. Shen A, Higgins DE (2006) The MogR transcriptional repressor regulates nonhierarchal expression of flagellar motility genes and virulence in *Listeria monocytogenes*. PLoS Pathog 2: e30.
5. Dove SL, Joung JK, Hochschild A (1997) Activation of prokaryotic transcription through arbitrary protein-protein contacts. Nature 386: 627-630.
6. Kuznedelov K, Korzheva N, Mustaev A, Severinov K (2002) Structure-based analysis of RNA polymerase function: the largest subunit's rudder contributes critically to elongation complex stability and is not involved in the maintenance of RNA-DNA hybrid length. Embo J 21: 1369-1378.
7. Deighan P, Diez CM, Leibman M, Hochschild A, Nickels BE (2008) The bacteriophage lambda Q antiterminator protein contacts the beta-flap domain of RNA polymerase. Proc Natl Acad Sci U S A 105: 15305-15310.
8. Deaconescu AM, Chambers AL, Smith AJ, Nickels BE, Hochschild A, et al. (2006) Structural basis for bacterial transcription-coupled DNA repair. Cell 124: 507-520.
